# Analysis of microRNA and Gene Expression Profiles in Alzheimer’s Disease: A Meta-Analysis Approach

**DOI:** 10.1038/s41598-018-20959-0

**Published:** 2018-03-19

**Authors:** Shirin Moradifard, Moslem Hoseinbeyki, Shahla Mohammad Ganji, Zarrin Minuchehr

**Affiliations:** National Institute of Genetic Engineering and Biotechnology (NIGEB), Tehran, Iran

## Abstract

Understanding the molecular mechanisms underlying Alzheimer’s disease (AD) is necessary for the diagnosis and treatment of this neurodegenerative disorder. It is therefore important to detect the most important genes and miRNAs, which are associated with molecular events, and studying their interactions for recognition of AD mechanisms. Here we focus on the genes and miRNAs expression profile, which we have detected the miRNA target genes involved in AD. These are the most quintessential to find the most important miRNA, to target genes and their important pathways. A total of 179 differentially expressed miRNAs (DEmiRs) and 1404 differentially expressed genes (DEGs) were obtained from a comprehensive meta-analysis. Also, regions specific genes with their molecular function in AD have been demonstrated. We then focused on miRNAs which regulated most genes in AD, alongside we analyzed their pathways. The miRNA-30a-5p and miRNA-335 elicited a major function in AD after analyzing the regulatory network, we showed they were the most regulatory miRNAs in the AD. In conclusion, we demonstrated the most important genes, miRNAs, miRNA-mRNA interactions and their related pathways in AD using Bioinformatics methods. Accordingly, our defined genes and miRNAs could be used for future molecular studies in the context of AD.

## Introduction

Alzheimer’s disease (AD) is one of the ultimately fatal neurodegenerative diseases affecting more than 35 million people worldwide^1^. In 2020, it has been predicted that the number of people affected with this disease will rise worldwide^2^. In developed countries, Alzheimer’s disease (AD) is the sixth leading cause of all deaths. While other major causes of death are declining, deaths caused by Alzheimer grows dramatically^3^. The neurodegenerative diseases such as AD is a type of disorder that neuronal function and structure is degenerated following by the death of neurons in the nervous system^4^. The greatest risk factor for neurodegeneration is age increase besides other risk factors. The pathophysiological process of AD patients before the clinical diagnosis is unknown^4^. The clinical pathogenesis of AD is said to be the accumulation of insoluble and extracellular amyloid-beta (Aβ) plaques and intracellular neurofibrillary tangles (NFT) in the brain^5^. This process has been shown intervened in long term potentiation (LTP), necessary in neuronal signaling, interfered in the signaling involved in pro-apoptotic signaling and results in neuronal loss^6^. The application of molecular mechanisms in the diagnosis of the AD is not clear, but it is thought that many factors are involved in the pathogenesis of AD. Therefore, such studies should have a high impact on AD diagnosis and treatment^3^.

miRNAs- single stranded non-coding RNAs- are small (18–25 nucleotides) and involved in the post-transcriptional regulation of gene expression^7^. Indeed, characterization of regulatory RNAs is one of the most important findings in the context of molecular biology in recent years^8^. In brief, miRNAs could bond partial complementarity to messenger RNA sequences, often in the 3′ untranslatable region (3′UTR). It has been shown that miRNAs participate and have implicated in neural development and differentiation as well as approximately 70% of all miRNAs expressions in the brain and they can function as biological regulators in neurons, for instance, neuronal differentiation, neurogenesis and synaptic plasticity^5,9^. Therefore, it seems that miRNAs have a potential role in neurodegenerative diseases,^5^ and in particular AD. Evidence showed that miRNAs are involved in deregulation of neurodegenerative diseases^4^. Many studies demonstrated the expression of specific miRNA in the central nervous system (CNS) with different roles^7,10^. Therefore a comprehensive study in miRNA’s involved in neurodegenerative diseases could be conveniently used in innovative therapies^4^.

The aim of this study is focused on miRNAs involved in AD and their target genes, the determination of the most important miRNAs, genes and their pathways in Alzheimer’s disease. Here we investigated and identified AD related differentially expressed genes (DEGs) and miRNAs (DEmiRs), miRNA-mRNA interactions and signaling pathways. Our results showed the different expressions of some miRNA and their target genes in a disease group *vs* normal; that this miRNA and their target genes have important roles in AD and other neurodegenerative diseases. In addition, pathways are related to miRNAs-target genes showed that it is significantly linked to the AD.

## Results

### Gene and miRNA expression profile

#### Differentially Expressed miRNAs and genes in AD

**Figure 1:**
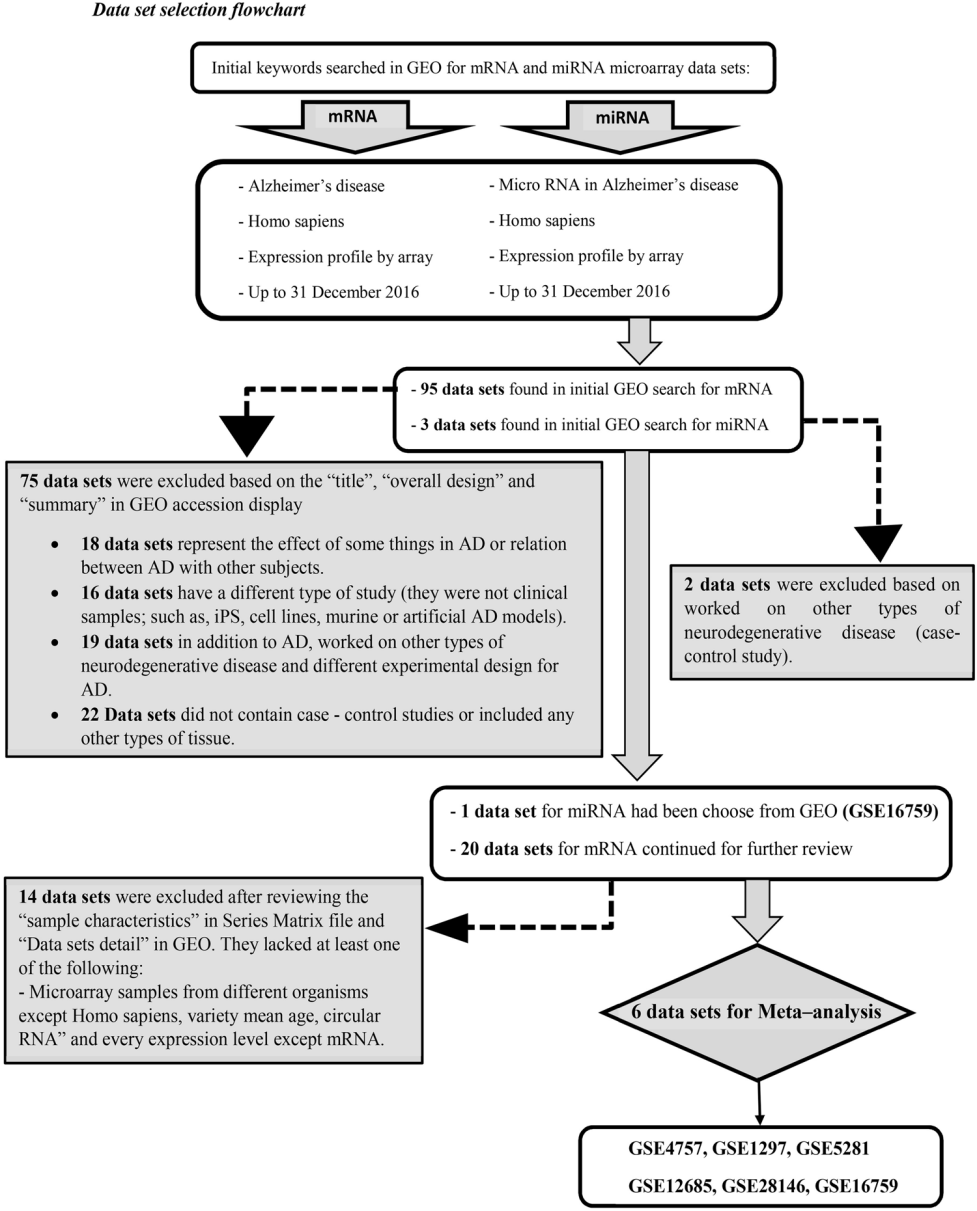
Data set selection flow chart. A total of 95 data sets from GEO was evaluated. Finally, 6 data sets for mRNA and 1 data set for miRNA were selected to be included in this meta-analysis.

**Figure 2:**
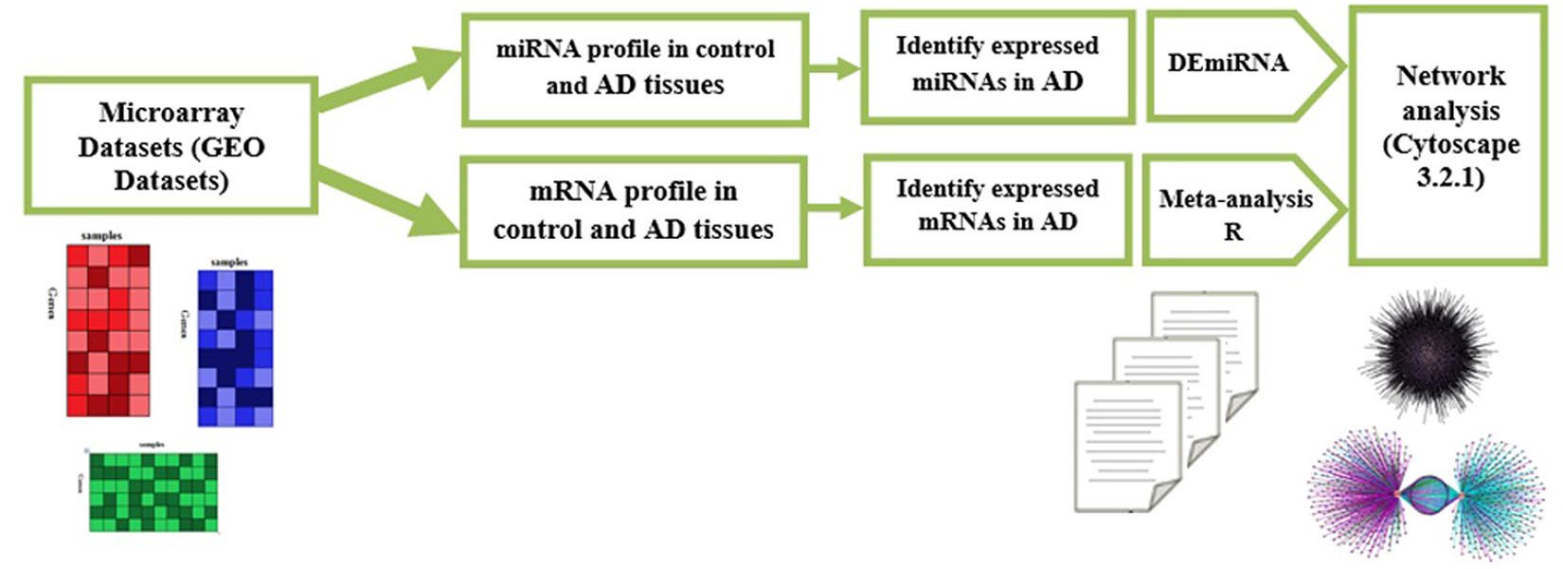
Workflow and analysis process.

Following the data sets selection according to our criteria (Fig. 1), analyzing the seven microarray data sets of genes and miRNAs expression profile according to the workflow (Table 1, Fig. 2) was done. After quality control and normalization, expression profile for each data set was created and a meta-analysis was performed. By using our criteria, differentially-expressed microRNA and genes were divided into Up- or Down-regulated miRNAs and genes. The P value < 0.05 and a fold-change ≥ 1.23 were set as the cut off values of DEgenes and DEmiRs. Our results in the meta-analysis showed a list of 1404 DEGs including 672 Up-regulated and 732 Down-regulated DEGs were selected and used in our subsequent analysis (supplementary Table S1). About DEmiRs expression analysis showed that a total of 179 differentially expressed miRNAs, 83 Down-regulated and 96 Up-regulated miRNAs in AD (supplementary Table S2). In Table 2 we summarized the most 30 Up- and Down-regulated DEmiRs which by their roles interfere in AD. For visualizing the Differentially Expressed miRNAs and genes, we sorted them. We also categorized the top Up- and Down-regulated DE genes in AD in Table 3.

**Table 1:** Microarray data sets used in this study and their experimental design. Gene Expression Omnibus dataset (GEO) is the one of the international public repositories in the context of high-throughput data; Controls: control samples; Patients: Alzheimer’s disease patients.

	GEO accession number	Experiment	Sample size		Platform
			Controls	Patients	
	**mRNA**				
1	GSE4757	Dunckley,…*et al*.^61^	10	10	[HG-U133_Plus_2] Affymetrix Human Genome U133 Plus 2.0 Array
2	GSE12685	Williams,…*et al*.^62^	8	6	[HG-U133A] Affymetrix Human Genome U133A Array
3	GSE28146	Blalock,…*et al*.^59^	8	22	[HGU133_Plus_2] Affymetrix Human Genome U133 Plus 2.0 Array
4	GSE1297	Blalock,…*et al*.^60^	9	22	[HG-U133A] Affymetrix Human Genome U133A Array
5	GSE5281	Liang,…*et al*.^63^	74	87	[HG-U133_Plus_2] Affymetrix Human Genome U133 Plus 2.0 Array
		Liang,…*et al*.^64^			
6	GSE16759	Nunez-Iglesias,…*et al*.^47^	4	4	[HGU133_Plus_2] Affymetrix Human Genome U133 Plus 2.0 Array
	**miRNA**				
7	GSE16759	Nunez-Iglesias,…*et al*.^47^	4	4	USC/XJZ Human 0.9 K miRNA940v1.0

**Table 2:** Top 30 DEmiRs obtained from microarray expression in AD patients. All the top miRNAs were ranked based on the P value. This table showed the miRNAs expression based on (≥ 1.23 fold-change) criteria and divided into Up- or Down-regulated miRNAs. FC: Fold Change.

Down-regulated			Up-regulated		
ID	P. Value	FC	ID	P. Value	FC
hsa-mir-12497|RNAz	0.003394	0.0263887	hsa-mir-19790|RNAz	0.0026067	3.4836785
hsa-miR-424	0.0011322	0.079282	hsa-mir-27120|RNAz	0.0011101	3.9197762
hsa-mir-44608|RNAz	0.0026307	0.0920438	hsa-mir-35456|RNAz	0.0032355	3.9345788
hsa-miR-181c	0.0015209	0.1018621	hsa-miR-122a	0.0029606	4.0567087
hsa-miR-301	0.0012602	0.1034073	hsa-miR-134	0.003466	4.499232
hsa-miR-656	0.0013878	0.1617726	hsa-mir-10939|RNAz	0.001997	5.3955761
hsa-miR-186	0.0025921	0.1711834	hsa-mir-10912|RNAz	0.0012295	5.7425097
hsa-mir-20546|RNAz	0.0013675	0.1773218	hsa-miR-617	0.0003481	6.1780417
hsa-mir-02532|RNAz	0.0031668	0.2263602	hsa-mir-30184|RNAz	0.0000352	6.3673885
hsa-mir-42448|RNAz	0.002298	0.2375547	hsa-mir-03996|RNAz	0.0031868	7.653148
hsa-miR-101	0.000485	0.2680839	hsa-miR-188	0.0004821	12.61189
hsa-miR-551b	0.0013837	0.2809868	hsa-mir-06383|RNAz	0.0010481	16.090057
hsa-mir-08570|RNAz	0.0019679	0.2865049	hsa-mir-23974|RNAz	0.0018586	23.14629
hsa-miR-29b	0.0014182	0.3212273	hsa-mir-40796|RNAz	0.0001212	25.918338
hsa-miR-189	0.0016735	0.4098583	hsa-miR-601	0.0013881	39.057145

**Table 3:** Top 30 differentially expressed genes (DEGs) identified in the meta-analysis of AD studies. All the top genes were ranked based on meta-analysis scores (this score calculated according to the P value of each gene in their datasets with R). Ave FC: average Fold change.

Gene symbols	Ave FC	Meta-analysis score	Gene symbols	Ave FC	Meta-analysis score
Down regulated			Down regulated		
*CARTPT*	0.615281653	4.06737E-06	*DYNLL1*	0.79243	0.000235
*ATP5F1*	0.77653477	4.03204E-05	*TMEM189*	0.760451	0.000258
*RNFT2*	0.65113094	6.13025E-05			
*USO1*	0.698005449	9.30377E-05	**Up-regulated**		
*COL5A2*	0.53826377	9.61705E-05	*GFAP*	1.856253	2.34E-06
*CALM1*	0.710120806	0.000113127	*TGFB1I1*	2.041183	2.05E-05
*DZIP3*	0.650845423	0.000127364	*APLNR*	1.912765	2.18E-05
*PLK2*	0.647124318	0.000137089	*ATP10A*	1.346906	3.42E-05
*BEX4*	0.69732923	0.000140728	*AQP1*	1.648286	0.000159
*MDH1*	0.694880723	0.000144727	*WAS*	1.323854	0.000162
*ATP2B3*	0.686082434	0.000156006	*DOCK2*	1.491173	0.000187
*ATP6V1E1*	0.721259284	0.000186009	*LRRFIP1*	1.420411	0.000192
*PFKM*	0.77010002	0.000211936	*IGFBP5*	1.399666	0.000198
*RAN*	0.640432884	0.000216575	*NAV2*	1.347951	0.000225
*CRH*	0.686953124	0.000220206	*FDFT1*	1.633492	0.000232
*MEST*	0.791186	0.000223	*MAFF*	1.4246	0.00027

**Figure 3:**
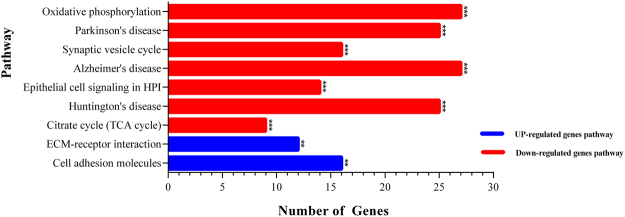
Top biological pathways and terms by ClueGo software in meta-analysis of Up- and Down-regulated genes in AD patients. The significant Up- and Down-regulated genes in AD patients were 672 and 732 different expression genes respectively that generated by meta-analysis and transferred into the ClueGO software (kappa score = 0.4). Pathways or terms of the associated genes were ranked based on the P value corrected with Bonferroni step down (*show the P value < 0.05).

**Figure 4:**
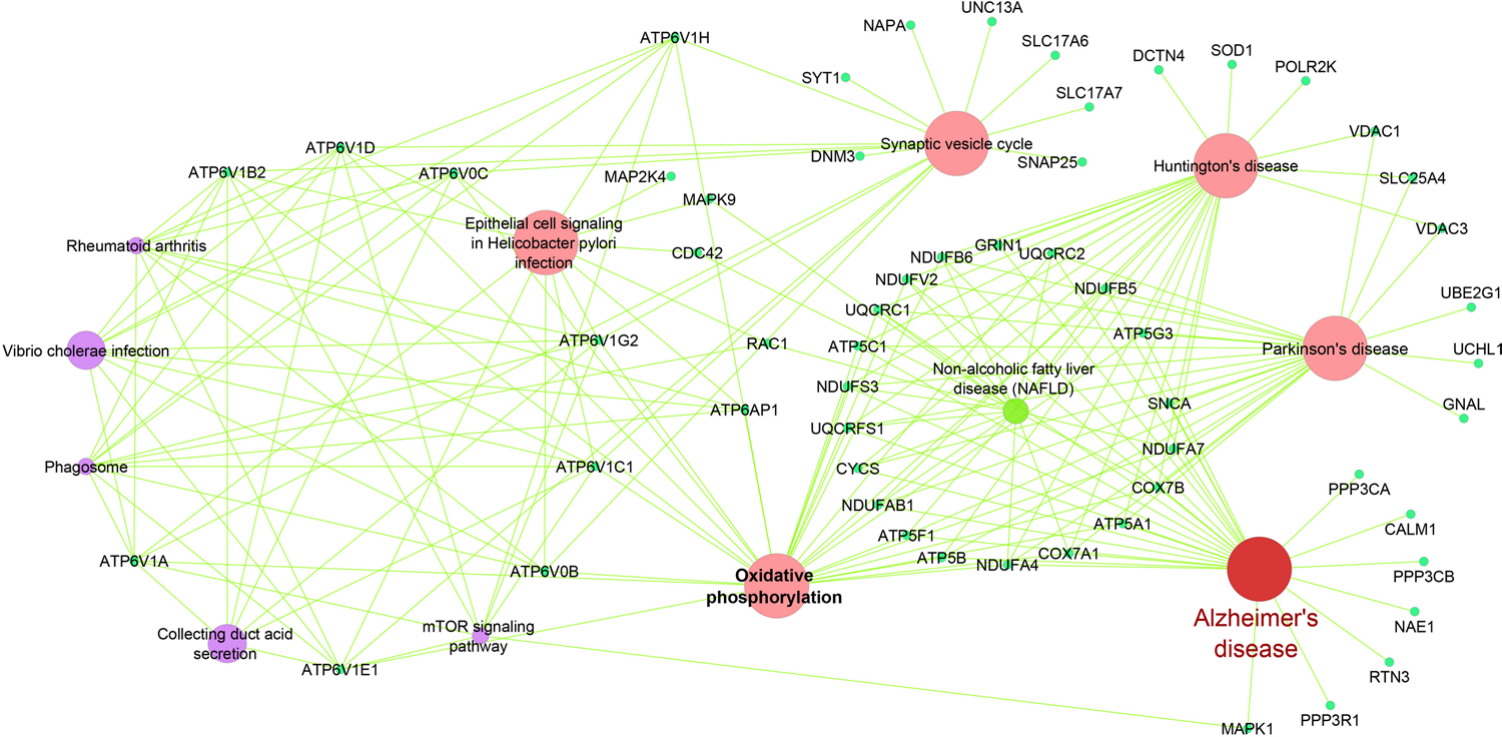
Alzheimer’s disease pathway and enriched gene ontology with Down-regulated genes. Among 10 significant pathways of Down-regulated meta-analysis genes, Alzheimer’s disease pathway and enriched gene ontology with other top significant pathways in this study have been visualized in CluGO software (kappa score = 0.4). It demonstrated the genes that are common among Parkinson’s disease, Alzheimer’s disease, and Huntington’s disease; also, the genes that are only involved in Alzheimer’s disease and other significant pathways.

**Figure 5:**
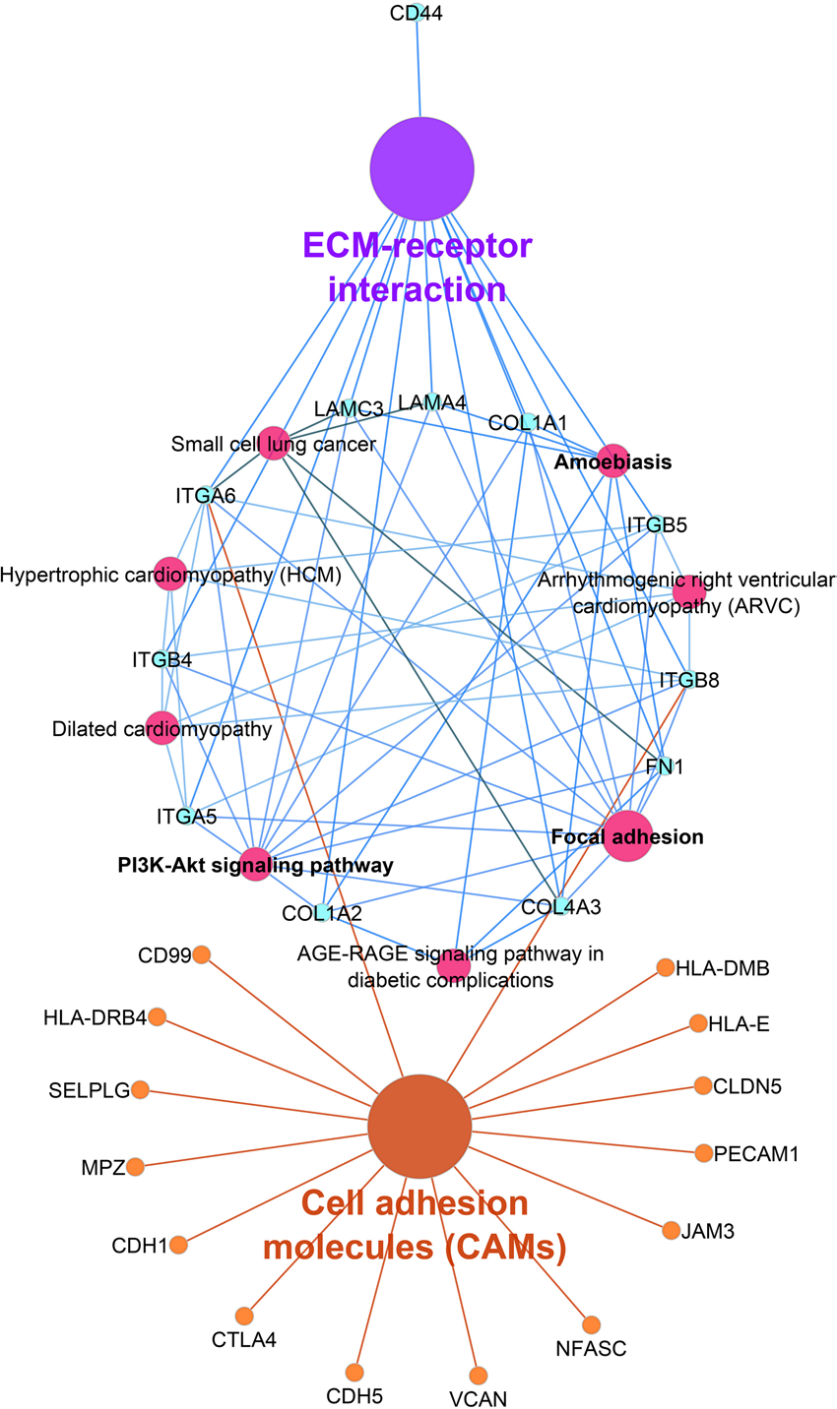
Enriched gene ontology and pathways that generated by Up-regulated genes in the meta-analysis. Enriched gene ontology and pathways have been visualized in CluGO software (kappa score = 0.4). Each node showed the biological process in their pathways. The color of each node represents the functional group that they belong to it. Edges demonstrate the gene-term and term-term interactions. Both significant pathways of Up-regulated meta-analysis genes, the ECM-receptor interaction and Cell adhesion molecules (CAMs) with involved genes have been shown in this pathway. The size of each node demonstrated its value in these pathways.

For further analyzing on the meta-analysis results (Up- and Down-regulated genes), we used ClueGO a Bioinformatics tool for clustering pathways and gene ontology terms. This tool visualized the interactions between genes and clusters; therefore, we can separate them easily based on their importance. The most important among them had been chosen by “Term P value Corrected with Bonferroni step down.” The KEGG pathway analysis of Up- and Down-regulated genes that were highly enriched have been shown here: the significant pathways in Up-regulated genes were ECM-receptor interaction and Cell adhesion molecules (CAMs) but for Down-regulated genes, which were involved in 10 pathways were oxidative phosphorylation, Parkinson’s disease, Synaptic vesicle cycle, Alzheimer’s disease, Epithelial cell signaling in *Helicobacter pylori* (HPI) infection, Huntington’s disease and citrate cycle (TCA cycle) were highly significant. These pathways and the number of genes involved, have been shown in Fig. 3 by individual curves (supplementary Tables S3, S4). The significant pathways of Up- and Down-regulated genes with their interactions have been visualized by ClueGo software in Figs 4, 5.

#### Study of brain regions and sex-related differences in gene expression in AD

**Figure 6:**
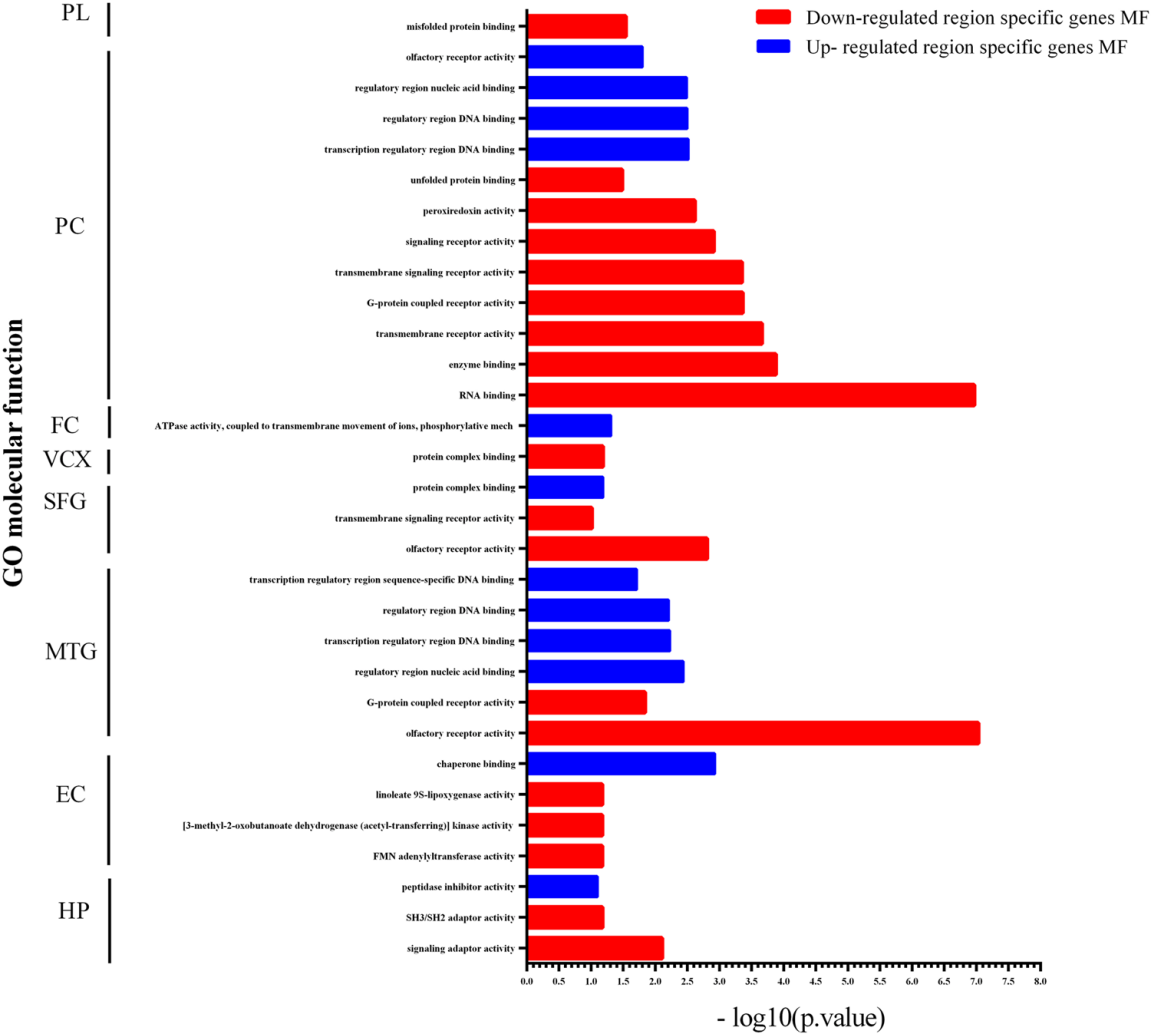
The molecular function of region specific genes in AD. Molecular function (MF) of all region specific genes in each brain region based on cut off P value < 0.001 have been selected. The significant MF for Up and Down-regulated region specific genes were shown. HP: hippocampus, EC: Entorhinal cortex, MTG: Medial temporal gyrus, PC: Posterior singlet cortex, SFG: superior frontal gyrus, VCX: primary visual cortex, FC: frontal cortex. PL: parietal lobe.

**Figure 7:**
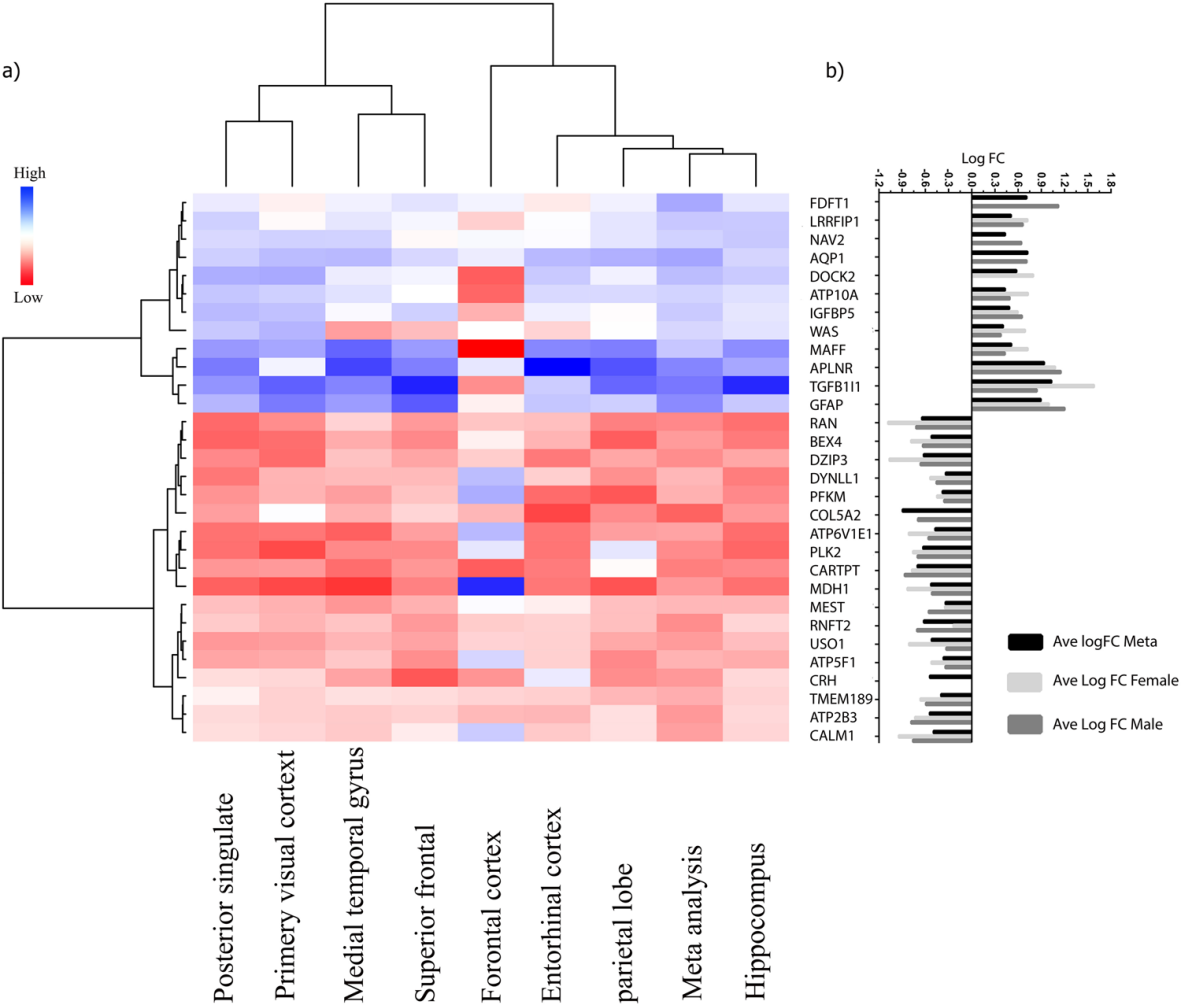
Comparing the gene expression pattern of top 30 genes from meta-analysis (**a**) in different brain regions (**b**) with Female and Male. (**a**) The heat map panel showed the top 30 differentially expressed genes in meta-analysis results versus different brain regions (posterior singlet cortex, primary visual cortex, medial temporal gyrus, superior frontal gyrus, frontal cortex, entorhinal cortex, hippocampus, parietal lobe *vs* DEGs in meta-analysis results). This panel compared the gene expression pattern in each type of brain regions between AD and control patients in six data sets (GSE12685, GSE4757, GSE5281, GSE1297, GSE28146, and GSE16759). The red color showed the low expression value, and blue color showed higher expression value. (**b**) This figure compares the LogFC of each gene among female, male and meta-analysis result. The Y-axis shows a selected top 30 gene names and the X-axis features black, light gray and gray bars which represent the LogFC by meta-analysis, Female and Male.

Analyzing the expression profile of brain regions with six data sets was done in which each data set represented one region in the brain and one of them (GSE5281) consists of more than one region; likewise, the sample size was insufficient for further studying of brain regions independently. Analyzing the gene expression profile on the six data sets according to brain regions separately and based on control *vs* AD was done. The sub meta-analysis of hippocampus and entorhinal cortex have been analyzed; but, the expression profile of other regions was utilized to continue the reviewing. The top DEGs, the top region specific genes and the genes which were in common with meta-analysis results in each brain region (based on the resold P value < 0.001) was categorized in supplementary Table S5. Also, the significant molecular functions of region specific genes (P value < 0.001) in eight brain regions were demonstrated in Fig. 6. The hierarchical clustering analysis was used for the top 30 DE genes in the meta-analysis result compared to the expression profile of all brain regions in Fig. 7a.

The sub meta-analysis of male and female with four data sets (GSE16759 due to the low sample size and GSE4757 due to its unknown gender was excluded) have been done. Our results in the context of sub meta-analysis on male and female showed the lists of 1903 DEGs in male (830 Up-regulated and 1073 Downregulated DEgenes) which 1210 genes were male specific and 2333 DEGs in female (1125 Up-regulated genes and 1208 Down-regulated DEgenes) that 1640 genes were female specific respectively (all details about gender specific genes and significant DEGs have been categorized in Supplementary Table S6). Thus, male and female specific genes were specified in AD; the female specific genes are involved in pathways associated with neurodegenerative diseases such as Oxidative phosphorylation, Alzheimer’s disease, Huntington’s disease, Parkinson’s disease pathways which demonstrated the importance of these genes in neurodegenerative including AD. In about male specific genes, no significant pathway was determined. Since, the aim of our study in this meta-analysis project was to exclude the interventive factors and categorized the top DEGs among AD across brain regions; for comparing the effects of gender on our meta-analysis result, we used T-test and one-way analysis of variance (ANOVA), these statistical analyses just among common DEGs of meta-analysis results, female and male was done; the outputs of statistical tests demonstrated that gender’s sub meta-analysis results do not have a significant difference in gene expression of the meta-analysis result (P value > 0.05). The top 30 DEGs in meta-analysis result which were common with female, male or either of them, based on LogFC have been visualized in Fig. 7b.

#### Investigating the gene expression profiles in AD with RNA-seq

In this study, we tried to select data sets, which as much as possible were matched and comparable with microarray sampling brain regions. Hence, studying on differential gene expressions between AD *vs* Control in three RNA-seq data sets (GSE53697 sampling from part of the dorsolateral prefrontal cortex (BA9), GSE67333 sampling from hippocampi brain regions and GSE57152 sampling from superior temporalis gyrus) have been analyzed^11,12^. The results demonstrated that the 1102 DEGs in GSE67333 (614 Up-regulated and 488 Down-regulated genes), 1032 DEGs in GSE53697 (324 Up-regulated genes and 708 Down-regulated genes) and 898 DEGs in GSE57152 (591 Up-regulated and 307 Down-regulated genes) with a threshold P value < 0.05 and FC ≥  1.23 that were categorized in supplementary Table S7. DEGs between RNA-seq and microarray data were analyzed. 95 common genes between the dorsolateral prefrontal cortex (BA9) and frontal cortex brain regions, 61 common genes on the hippocampus in RNA-seq and microarray data sets; also, 240 genes between superior temporalis gyrus and medial temporalis gyrus in RNA-seq and microarray data sets respectively have been obtained (P value < 0.05). The highly significant common Up- and Down-regulated genes between RNA-seq and microarray have been categorized in Table 4.

**Table 4:** The top significant common genes between RNA-seq and microarray. According to cut off P value < 0.05 and FC ≥  1.23. The common and top significant Up- and Down-regulated genes in GSE53697 (sampling from BA9 which part of the dorsolateral prefrontal cortex (DL-PFC)), GSE67333 (sampling from hippocampi) and GSE57152 (sampling from superior temporalis gyrus) with microarray results which consist of submeta analysis of hippocampus, expression profile of frontal cortex and medial temporal gyrus, have been shown.

	Up-regulated					Down-regulated				
	Gene Symbol	RNA-seq		microarray		Gene Symbol	RNA-seq		microarray	
		logFC	P value	logFC	P value		logFC	P value	logFC	P value
Common genes in FC and BA9 of DL-PFC	*NRN1*	2.2334	0.0198	0.8070	0.0213	*PRELP*	−3.9582	0.00005	−0.3136175	0.0099
	*GPRASP1*	1.5928	0.0209	0.9407	0.0354	*EPAS1*	−2.1622	0.000205	−0.39160792	0.0159
	*ESRRG*	1.3135	0.0250	0.2804	0.0228	*KIF1C*	−3.0637	0.001339	−0.44908542	0.0246
	*TPBG*	1.6981	0.0252	0.5743	0.0011	*FKBP10*	−1.9021	0.001747	−0.29188125	0.0483
	*KCNK1*	1.4959	0.0252	0.6657	0.0004	*KLF4*	−3.3412	0.002665	−0.3612275	0.0067
	*HTR2A*	1.9082	0.0253	1.3014	0.0007	*NQO1*	−3.7408	0.003207	−0.31109333	0.0476
	*GSTO1*	1.7010	0.0261	0.6422	0.0087	*ELN*	−2.1514	0.003593	−0.3799525	0.0417
	*TOMM20*	1.7740	0.0301	0.6976	0.0026	*FBLN1*	−1.8663	0.007021	−0.33266417	0.0118
	*GABRA1*	2.4678	0.0303	1.2669	0.0001	*SOX21*	−1.9109	0.012743	−0.39442583	0.0059
	*OXR1*	1.7054	0.0308	0.6058	0.0051	*SMOX*	−1.4258	0.015775	−0.41022292	0.0495
Common genes in Hippocampus	*PRKCQ-AS1*	3.6348	0.00005	0.4930	0.0337	*GLS2*	−2.4687	0.00005	−0.4732	0.0467
	*TRIM16L*	0.9125	0.00005	0.7303	0.0295	*SLC22A24*	−2.4552	0.0006	−0.3981	0.0043
	*PLK5*	1.8366	0.0001	0.9223	0.0271	*PDXDC1*	−4.8043	0.001	−0.2931	0.0144
	*CNOT1*	2.4604	0.0007	0.3935	0.0049	*MYH16*	−0.7303	0.0010	−0.7485	0.0449
	*LINC00648*	3.7324	0.001	0.3031	0.0224	*ZNF704*	−3.4464	0.0011	−0.3069	0.0272
	*APOL4*	1.6363	0.0010	0.4707	0.0166	*DEFB108B*	−1.9448	0.0045	−0.7843	0.0343
	*APBB1IP*	2.6487	0.0011	1.0084	0.0081	*CDC42SE2*	−1.8370	0.0083	−0.4003	0.0001
	*LINC00700*	1.9264	0.0014	0.5462	0.0453	*DNAH14*	−0.5588	0.019	−0.6766	0.0079
	*STRAP*	2.3408	0.0018	1.0526	0.0248	*DNAJC13*	−2.1886	0.0205	−0.4092	0.0464
	*NUMA1*	2.1248	0.0020	0.7587	0.0088	*KCNK10*	−0.4884	0.0226	−1.1787	0.0216
Common genes in STG and MTG	*C9orf3*	4.61003	0.00005	1.364899	0.01602	*CRYBB2P1*	−4.42879	0.00005	−0.81855005	0.03695
	*CPNE3*	3.3581	0.00005	1.1969213	0.0243	*FAM208A*	−4.10653	0.00005	−0.4882346	0.00118
	*GLUL*	2.82662	0.00005	1.393713	0.0218	*KNTC1*	−3.7896	0.00005	−1.6199	0.0109
	*IKBKB*	2.82657	0.00005	1.240875	0.0020	*NAT9*	−4.2662	0.00005	−0.6446	0.0109
	*PFKFB3*	3.89407	0.00005	2.198218	4.49E-07	*NRG4*	−5.5145	0.00005	−1.1152	0.0405
	*SLC11A2*	2.16711	0.00005	1.957787	0.000037	*SETD5*	−2.676	0.00005	−0.8030	0.0046
	*SPP1*	3.2241	0.00005	0.9801302	0.0206	*TMCC1*	−2.63472	0.00005	−1.098779	0.008445
	*USP40*	2.10273	0.00005	1.4242945	0.00124	*TNKS*	−2.05614	0.0002	−0.8721588	0.027795
	*ZCCHC7*	5.67435	0.00005	1.0294421	0.00326	*ZBTB45*	−4.02681	0.0003	−1.16852	0.024
	*ZNF528*	3.51179	0.00005	0.8404265	0.0287	*NAA38*	−3.54314	0.00035	−0.92694	0.0019665

#### Gene-miRNA regulatory network in AD

**Figure 8:**
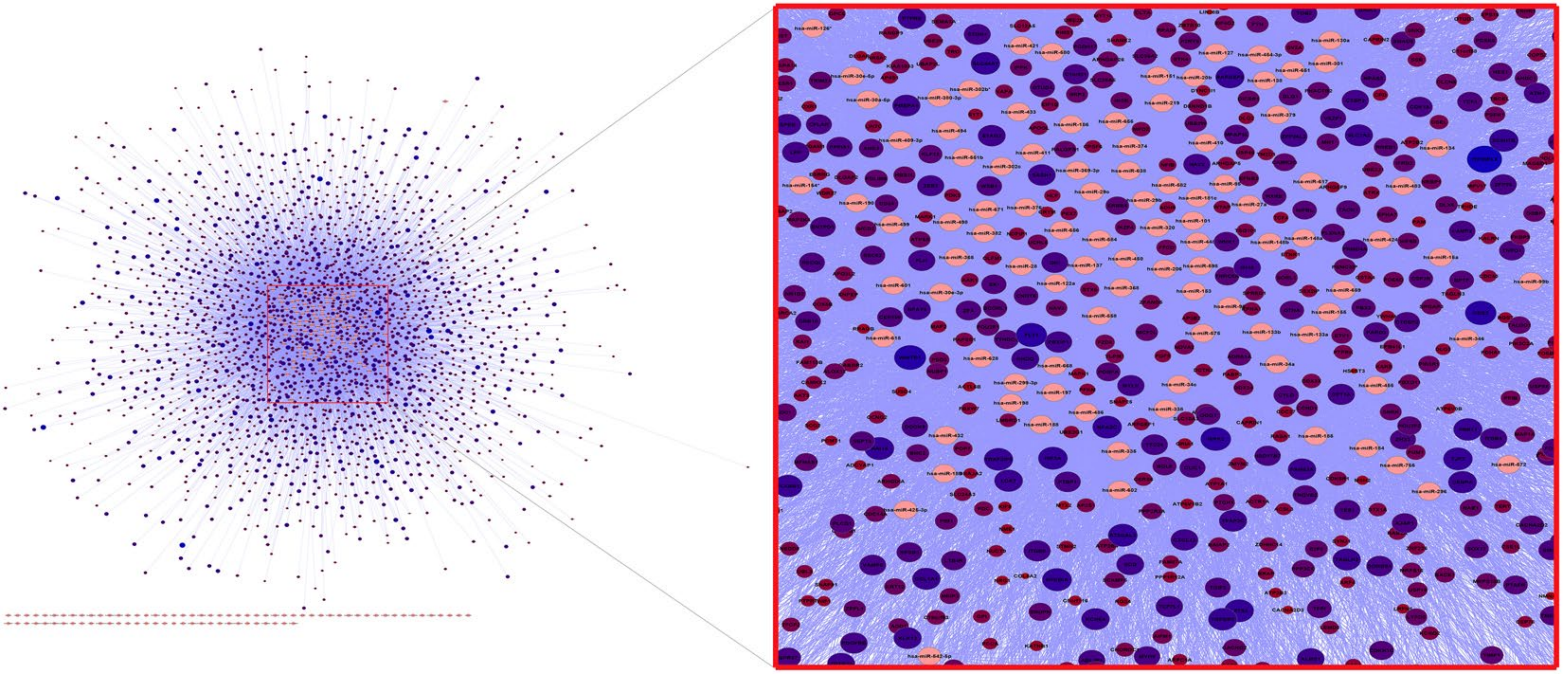
The regulatory subnetwork in AD that showed the DEmiRs and their target genes. The differential expression has been shown by node color gradient. The pink nodes represent miRNAs, but a gradient red to blue colors showed Down-regulated and Up-regulated target genes respectively.

Our results demonstrate the different expression value for each type of genes, so we could divide the Up- and Down-regulated genes. All of these meta-analysis results and miRNAs were transferred into the Cytoscape 3.2.1, and the network was constructed. The regulatory network constructed by cyTargetLinker^13^ using three databases (TargetScan, MicroCosm V5, mirTarbase). The regulatory miRNA-target genes network included 18189 genes, 181 miRNA, and 113796 edges. We mapped the expression data that included meta-analysis result and DEmiRs to the regulatory network; we filtered the target genes that were not included in our meta-analysis and did not have an expression data then based on expression value, the node size and color change. This regulatory network included 2438 genes, 181 miRNA and 16886 edges that were differentially expressed in AD (Fig. 8).

#### Construction of Gene Regulatory subnetwork for miRNA-target genes

**Figure 9:**
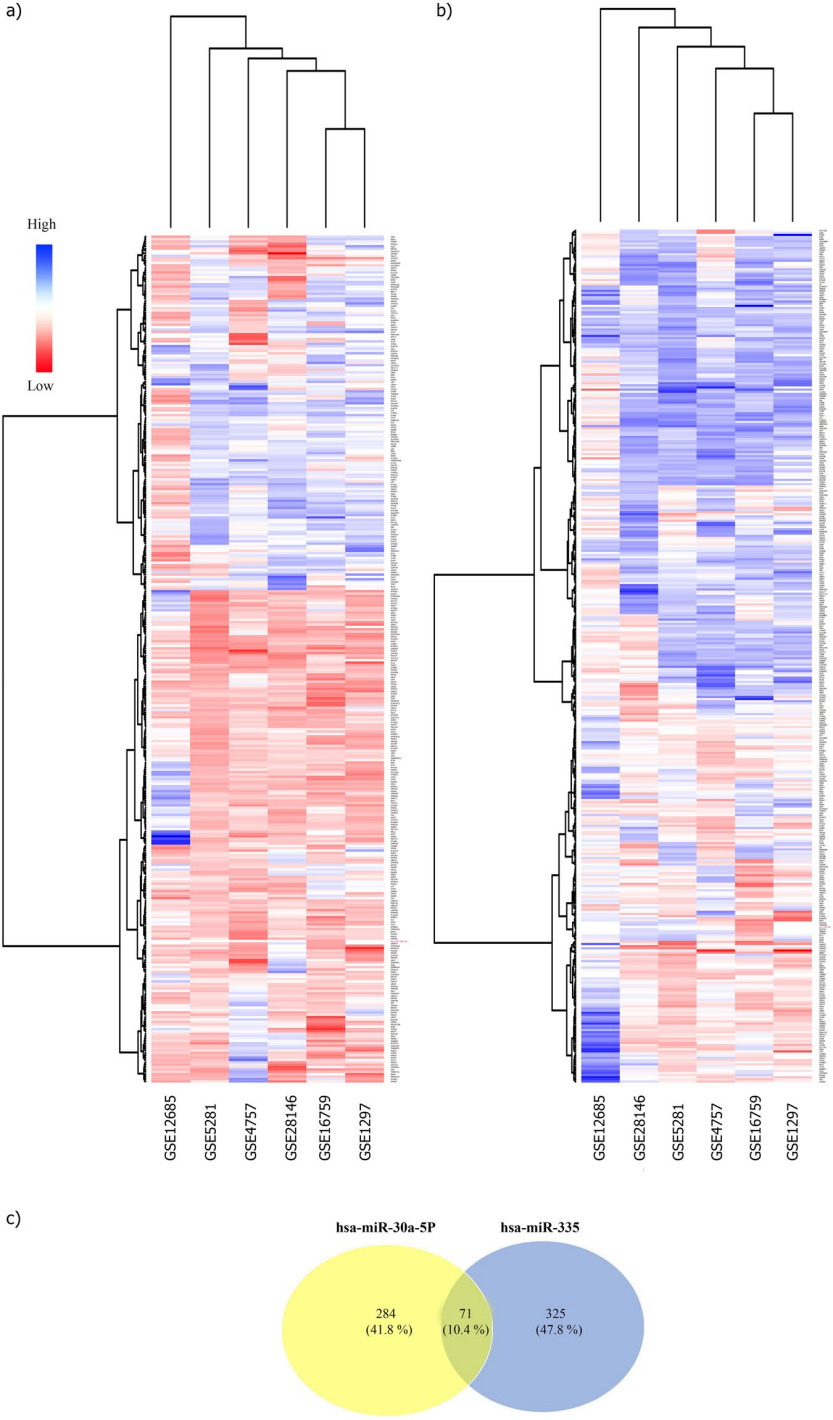
The cluster heat map of miR-30a-5p and miR-335 with their Venn diagram. Differentially expressed microRNAs (DEmiRs) hub in network and genes that were targeted of it. The red color showed the low expression value, and the blue color showed higher expression value. (**a)** The miR-30a-5p with expression profile of its target genes. (**b)** The miR-335 with expression profiles of its target genes. (**c)** This panel showed the overlap between miR-30a-5p and miR-335 target genes; 71 genes are common between both miRNAs.

Surveys conducted by centiscape^14^ showed our network hubs were miR-30a-5p and miR-335 which regulated most genes involved in AD in our meta-analysis results. Subsequently, hierarchical clustering analysis was done in order to find significant differences in both differentially expressed miRNAs and genes expression between the AD and Control groups for the hubs of our network - miR-30a-5p and miR-335 (Fig. 9a,b). Each hierarchical cluster for miR-30a-5p and miR-335 demonstrated the expression profile of near neighbor target genes. Furthermore, Venn diagram by VENNY 2.1 tool^15^ has been drawn for common genes between both miRNAs (Fig. 9c). The results showed that 71 genes are common target genes for both miRNAs.

**Figure 10:**
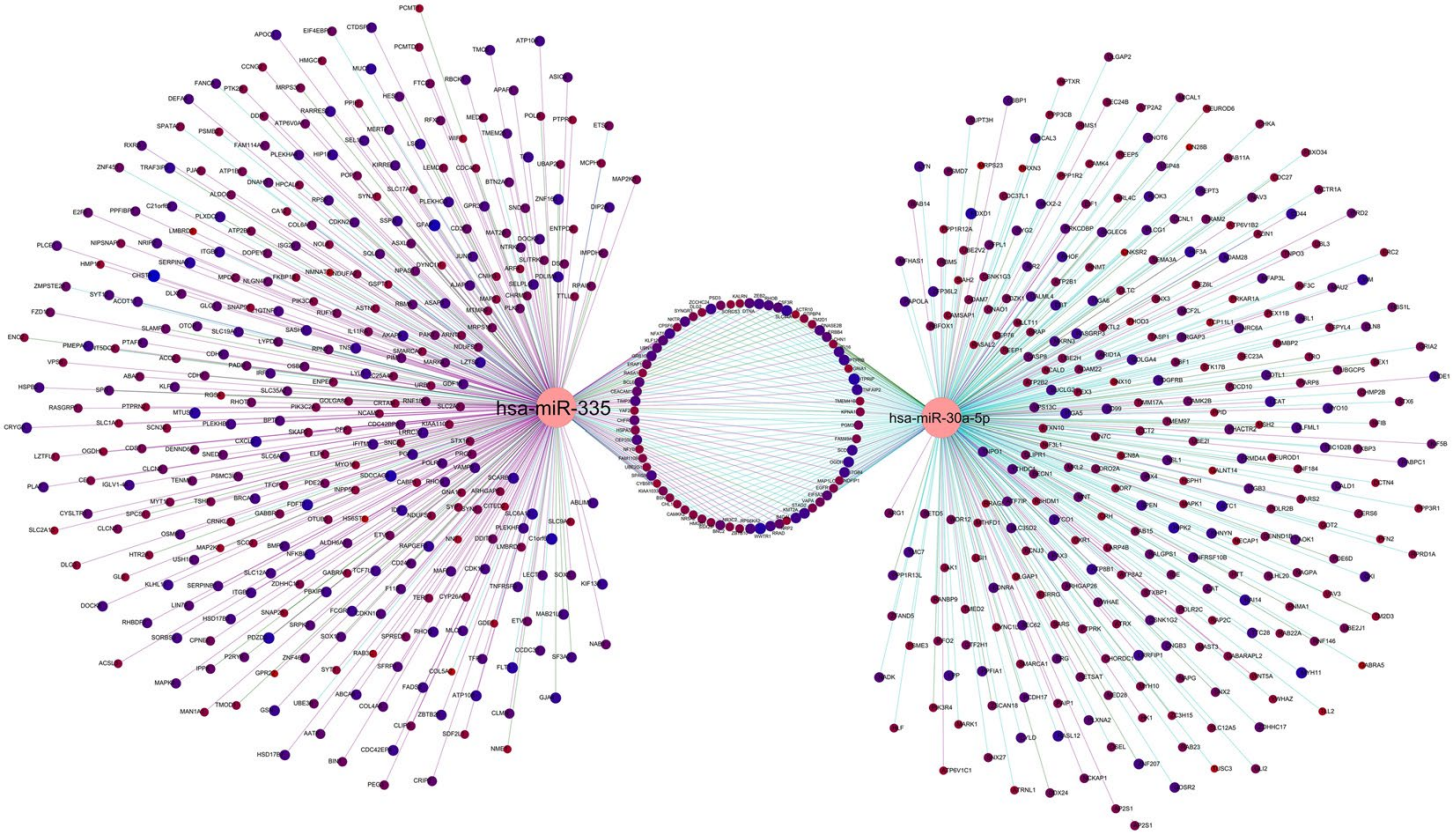
The near neighbor nodes subnetwork of miR-30a-5p and miR-335. This subnetwork represented the near neighbor target genes of miR-30a-5p and miR-335 with their common genes. The node size and color showed the expression value (blue color; big node showed the higher expression value and red color; small node showed the low expression value), and the edges color demonstrated the source of each target gene prediction. TargetScan: indigo, MicroCosm V5: green, mirTarbase: Violet.

Expression of miR-30a-5p (logFC −0.61128) and miR-335 (logFC −0.91861) decreased, but both have Up- and Down-regulated target genes. miR-30a-5p have 355 near neighbor target genes that 135 genes with positive logFC and 220 genes with negative logFC, for miR-335 among 396 near neighbor target genes have 213 positive logFC and 183 negative logFC genes in AD. The circulate subnetwork of miR-30a-5p and miR-335 with immediate neighbors had been constructed by Cytoscape 3.2.1, here we demonstrated the most important target genes. The node size and color (red-blue) represented the expression value of Down- and Up-regulated respectively. Also, the edges’ colors showed the source of prediction target genes (Fig. 10). The validated target genes of miR-30a-5p and miR-335 *vs the* predicted ones have been categorized in supplementary Table S8.

#### KEGG pathway Analysis of miRNA-335 and miRNA-30a-5p target genes

**Figure 11:**
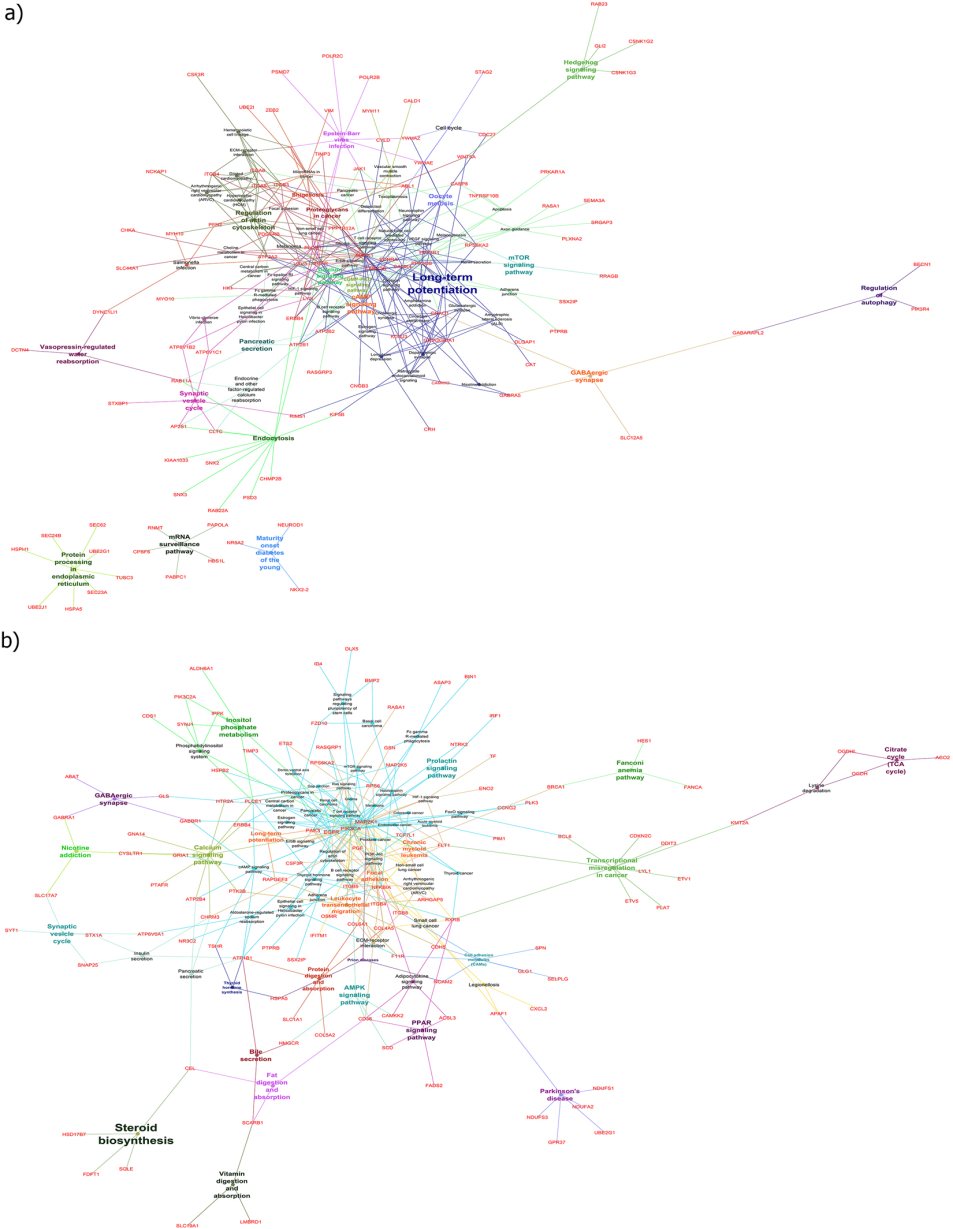
Functional analysis of target genes (**a**) miR-30a-5p (**b**) miR-335 in KEGG pathway by ClueGo software in Cytoscape 3.2.1. The results of centiscape analysis elicited from the gene regulatory network and then the target genes of those hub nodes transferred into CluGo and were grouped with it as a functional cluster. Each node represents a KEGG pathways process, and their associated genes are represented as dots. All the nodes and their colors showed the functional group to which they belong. Edge of this functional analysis demonstrated the term-term interactions or term-genes interactions. The enrichment significant term showed by the size of each node.

According to a KEGG pathway^16–18^ analysis of ClueGo plugging software, we demonstrated the significant functions and pathways of near neighbor target genes of miR-30a-5p and miR-335. All of them sorted based on the Bonferroni step-down (Table 5). Also, visualized by CluGo in Fig. 11a and b. Our results represented the significant pathways of miR-30a-5p near neighbor is Long term potentiation (LTP), for miR-335 the near neighbor target genes interfered in the Steroid biosynthesis pathway but the role of this pathway was not significant.

**Table 5:** The significant pathways of miR-30a-5p and miR-335 near the neighbor target genes in AD. For miR-30a-5p, Long-term potentiation is the pathway that near neighbor target genes interfered in that with the significant P value. But for miR-335, it is not a significant pathway. Here we showed the target genes are involved in each pathway.

**miRNA**-30a-5p near neighbor nodes			
GO ID	GO Term	Term P value Corrected with Bonferroni step down	Associated Genes Found
KEGG:04720	Long-term potentiation	0.00756605	*CAMK2B*, *CAMK4*, *GRIA1*, *GRIA2*, *MAPK1*, *PPP3CB*, *PPP3R1*, *RPS6KA2*
**miRNA-335 near neighbor nodes**			
KEGG:00100	Steroid biosynthesis	0.105889168	*CEL*, *FDFT1*, *HSD17B7*, *SQLE*

## Discussion

Alzheimer’s disease is a general neurodegenerative disorder and its pathophysiological process mostly begins before clinical diagnosis. During the pre-clinical process most AD patients show no symptoms believed is^19^. Therefore, molecular study of AD could have an important role in detecting genes involved in the disease. On the other hand, a variety of researchers showed that miRNAs and expression of target genes were tightly associated with molecular events in neurodegenerative diseases such as AD^4^. The miRNAs in the central nervous system have been shown that contributes to the regulation of development, survival, function, and plasticity^20^. Moreover, any disruption and alterations in microRNAs and their expression in neurons, leading to neurodegenerative diseases such as Alzheimer’s disease (AD)^20^. It is noteworthy that miRNAs have a high abundance in the central nervous system, and mostly their expression patterns are brain-specific^21^. According to our results, the DEGs and DEmiRs both can be a potential candidate for biomarkers in AD. Other studies such as qRT-PCR can also be suggested for confirming them.

Differentially expressed genes and their relation to sex have been acknowledged in some publications^22,23^. Albeit, it is very important to know that there is a difference between male and female in the brain structure and function; but, sex-related differences in gene expression are depended on the brain tissues and age^22^. A large number of genes are varied in the developmental stages of the brain^24^; this is due to the majority of brain development and organization and the influence of gender occurring in adulthood^25^. The risk of AD is depended on age and gender that in women is higher than the men. Among the proposed reasons for this difference, the mitochondria and hormonal disfection^26,27^ could be mentioned, where, hormone therapy and estrogenic compounds worked as a protection in front of the amyloid-beta toxicity^26^. Based on our results there are more than one hundred genes which specifically expressed in male and female and the other possible mechanism for gender response against AD could be resulted from their associated pathways. Meanwhile, in this study, we found the significant pathways with female specific genes which are related to AD and indicated their role and function in this neurodegenerative disease. The statistical analysis on the common genes between male, female and meta-analysis demonstrated that there are no significant differences between the gender and meta-analysis results. Therefore, the common genes between meta-analysis, and sex could represent the new window of study in AD.

In our study on DE genes of brain regions, we analyzed eight brain regions (posterior singlet cortex, primary visual cortex, medial temporal gyrus, superior frontal gyrus, frontal cortex, entorhinal cortex, hippocampus, parietal lobe) based on comparing the DEGs in each brain region and meta-analysis. The molecular function (MF) of top Up- and Down-regulated region specific genes showed that their function in some cases was in common among SFG, VCX, PC, and MTG. The most significant MF, among region specific genes were olfactory receptor activity and RNA binding (Fig. 6). Studies revealed that olfactory receptors (ORs) have been expressed in the human brain regions and the gene expression patterns have altered in several neurodegenerative diseases such as PD and AD. The differentially expressed OR genes (Up- or Down-regulated genes) have been reported in AD^28^. In our results, we demonstrated the expression of top genes which are related to these MF and played their function in each brain region. Also, aggregation of RNA binding proteins in the cytoplasm under stress conditions produced the stress granules (SGs). Accumulation of these SGs in the brain have a pathological impact in AD and other neurodegenerative diseases; due to altered the normal physiological activities of RNA binding proteins^29,30^.

As far as we are concerned, our research categorized the top and significant DEGs in the different brain regions and compared and analyzed the results using a meta-analysis output. Our findings can be helpful for understanding the most important DEGs in AD and making a connection between the gene expression level and higher level information about their functions, interactions and pathways. In the further study, we would like to analyze the region specific genes by using more data sets with a high sample size that are specialized on each specific brain region. These will increase the accuracy and will avoid false positive data in the study.

Studying on the RNA-seq data in AD and on the three parts of brain regions (part of the dorsolateral prefrontal cortex, hippocampus and superior temporalis gyrus) demonstrated the most important DEGs. The difference in brain regions sampling and low replication on the RNA-seq data rather than the microarray results can be a contributing factor in reducing the common genes between these two techniques. Although, microarray meta-analysis is still a widely used method in the context of gene expression, but the analysis of RNA-seq data beside to the microarray results can lead to heighten the accuracy of these results^31^. Due to the lack of sufficient RNA-seq data for all brain regions on Alzheimer’s disease, we did our study on three brain regions and compared the outputs with hippocampus submeta analysis, the expression profiles of frontal cortex and medial temporal gyrus. Eventually, the genes which were Up-or Down-regulated in this brain regions have been reported.

Our analysis reports have shown the significant pathways between Up-regulated genes already linked to AD: i) The ECM-receptors and their ligands as a molecular function, play a significant role in neuronal development and synaptic activity^25^. The ECM receptors and cell adhesion molecules (CAMs) participate in cellular interactions, which are involved in major neurodegenerative diseases such as AD. Also, ECM proteins were up-regulated in AD and during the aging^32^; hence, we demonstrated these pathways with their related DE genes which are Up-regulated in AD. ii) The CAM pathway, which has a central role in the neuronal cell adhesion and has a critical function for the synaptic formation and blood-brain barrier integrity for neurotransmission. In AD, loss of the synaptic pathway is the strongest impairment that has been done. In addition, a different expression of cell adhesion genes mainly seen in AD and Parkinson disease^33,34^.

The significant pathway results between Down-regulated meta-analysis genes have demonstrated which Down-regulated genes are involved in AD i.e.: Oxidative phosphorylation (OXPHOS), Parkinson’s disease, Synaptic vesicle cycle (SVC), Alzheimer’s disease, Epithelial cell signaling in *Helicobacter pylori* (HPI) infection and Huntington’s disease were the top involved pathways. In recent studies, this subject has been confirmed that alterations in the function of Oxidative phosphorylation (OXPHOS) have involved in the pathogenesis of AD. Also, the toxic effect of Aβ and tau protein on the OXPHOS cause the decreased neuronal survival^35^. Therefore, alterations in function OXPHOS can increase the risk of AD.

The Epithelial cell signaling in *Helicobacter pylori* infection are other pathways associated with AD. Based on AD different subtypes-levels of Aβ, tau hyperphosphorylation, ubiquitination and *etc.,*- inflammation and immune processes has displayed the different roles since Alzheimer disease patients which use the nonsteroidal anti-inflammatory drugs (NSAIDs) reduce the risk of Alzheimer disease^36^. Therefore, immune and inflammatory pathways play an important role in modulators of AD^36^. It can also be inferred that epithelial cell signaling in *Helicobacter pylori* infection has an effect on AD development it is noteworthy that hyperphosphorylation of tau protein is associated with defects in neurons or the loss them^37^. Infection with Helicobacter pylori (H. Pylori) which is a gram negative bacterium, is related to the AD^37^. Hyperphosphorylation of tau protein is one of the events seen in the AD patient’s brains^37^. Studies on the AD patients and normal people showed the significant H. Pylori and anti-H. Pylori IgG antibodies have been observed in cerebrospinal fluid (CSF) and their serum^38,39^. *H*. *Pylori* induced the tau phosphorylation in the AD special sites^36^ so, it is effective in occurring AD. Here we showed the involvement genes in each pathway with the expression value.

Alzheimer’s disease (AD), Parkinson’s disease (PD) and Huntington’s disease (HD) are important neurodegenerative disorders, and their involved genes mostly overlap. It is noteworthy that there are factors in common between AD and PD. A common and major neuropathological alteration, for instance, a decline in locus coeruleus (LC) noradrenergic neurons occur in AD and PD, that leads to progression of both of these disorders^40^. In addition, AD and PD have different clinical and pathological features but display the overlap molecular mechanisms^41^. Huntington’s disease (HD) is dominantly inherited and occurs in the 4th to 5th decade of life, but the AD is an aging disease and happens in older age. Albeit, it is mentioned that HD increases the risk of AD among elderly HD patients and demonstrated the co-occurrence of both of them^42^. The role of SVC in neurodegenerative diseases is specified and any changes in this pathway contributed in the development of diseases^43,44^. In about the TCA cycle pathway, any alteration in (TCA) cycle enzymes of mitochondria and changes in its activity may be critical to increase the risk of AD. Therefore, studying on TCA cycle will improve our understanding the mechanisms and will be effective for AD patients^45^. Therefore, our results categorized the genes that are specific or common for each or both of them. likewise, For all of the significant pathways, we categorized the involvement genes (Up- and Down-regulated) that will be crucial for continuous researchers and other types of detection or diagnose in AD patients. In the second part of our analysis, we focused on the miRNAs and target genes that are most important in AD. Centiscape results according to the degree and betweenness of our network were miR-335 and miR-30a-5p which had the most score and were the regulators in AD network. The expression of both of them decreased so, subnetworks were constructed with near neighbor nodes of miR-30a-5p and miR-335, which were the hubs of AD network analysis. miR-30a-5p with 356 and miR-335 with 397 neighbor nodes were the most interacted nodes respectively.

Based on our expression analysis on miR-30a-5p and miRNA-335 they have been decreased. The expression of miRNAs are different in each part of a brain. Mark N. Ziats *et al*. demonstrated in 2014 that miRNAs in the dorsolateral prefrontal cortex differentially expressed rather than other parts of the brain, such as the cerebellum, hippocampus and other regions of the prefrontal cortex^46^. On the other hand, in our study, the miRNA microarray data set had been sampling from the parietal lobe of postmortem of Alzheimerian patient’s brains^47^ so the different types of expression are perhaps because of the sampling regions. miR-30a-5p and miR-335 are one of the miRNAs that are involved in the AD, and their expression decreased in this brain region of AD patients. Wang-Xia Wang *et al*. at 2011 studied the miRNA expression profile in gray and white matter showed miR-335 and miR-30a-5p have been Down-regulated in a gray matter of AD patient^48^. Also, it is worthwhile to say miRNA often could be co-expressed with its targets^21^. In AD the main cause of the synaptic disfunction is the aggregation of amyloid-β (Aβ) peptides in the neuronal system which leads to a disarrangement, and cell loss^49^. The KEGG pathway analysis of miRNA30a-5p and its first neighboring nodes have been sorted by the Term P value corrected with step down showed that Long-term potentiation (LTP) is the significant pathway for all the near neighbor targets but about miR-335 we could not find any significant pathway.

The KEGG pathway analysis on the miRNA 30a-5p near neighbor target genes demonstrated that among all of them, CAMK2B, CAMK4, GRIA1, GRIA2, MAPK1, PPP3CB, PPP3R1, RPS6KA2 genes are significantly involved in LTP. Studying in the hippocampal LTP demonstrated its association with learning and memory. So, in AD LTP is flawed because of accumulation of Aβ and other fragments^50^.In neuroscience, LTP is the chemical transmission function and synaptic activity between two neurons^51^. The expression of CAMK2B and RPS6KA2 genes increased, but CAMK4, GRIA1, GRIA2, MAPK1, PPP3CB, PPP3R1 genes decreased in our meta-analysis study on AD.

The present study prepared and categorized genes and miRNAs in AD based on available microarray and RNA-seq data sets. Therefore, the reliable sources, which have been proposed in this study, are the most important ones. Hence, we faced with some inevitable technical limitation. Some of these limitations which are mentioned here: Microarray is one of the most powerful technique for analysis of gene or protein expression simultaneously; but, this technology has several limitations. The hybridization of probes to bind to a target sequence and specificity of them with great importance, but cross-hybridization in this context will diminish the specificity. Albeit, determining the four levels of hybridization specificity in the context of microarray hybridization could be effective but low hybridization specificity in each of which four level might be occurred. Therefore, specificity of hybridization in microarray is one of the critical limitations of this technique^52,53^. Using microarray data sets, which were obtained from multiple studies in different conditions, increased the heterogeneity in our study. In addition, the low sample size and unknown characteristic details (for instance, gender, ethnic, age, *etc.)*, different quality and amount of RNA are the major challenge in microarray analysis. However, we tried to minimize the effects of this limitation where we doing late integration meta-analysis and utilizing appropriate methods for analyzing data. Quality control and normalization is the critical content in microarray analysis and will decrease the false data in microarray analysis. As already, mentioned microarray is one of the most powerful techniques to evaluate the gene or protein expression. Also, according to differentially expressed genes in the microarray, usually we have a widespread differentially expressed genes, for instance, gene expression with very low expression value but insignificant or modest DEGs with significant values. Filtering microarray data is an essential factor are mostly assigned to cut off. In about RNA sequencing technique, we have a limitation in the context of the depth of sequencing and the number of replications that are important factors for demonstrating the reliable DEGs. Meanwhile, due to the cost of this method, considering the depth of sequencing and the number of replications in the appropriate condition is very difficult and usually, one of them is being investigated. On the other hands, the different sequencing methods in RNA-seq analyzing produced various DEGs output. Thus, All of them should be considered^54^. Nevertheless, RNA-Seq is a new technique and under development and despite the cost, widely used in the context of gene expression^55^ and when it studied with microarray, they can be complemented with each other^56^.

In conclusion, our finding suggests that the different expressions of genes and miRNAs are one of the most important variables in AD. Bioinformatic Analysis could help us to find the most important genes, miRNA, miRNA-mRNA interactions and their related pathways. Hence, these should be probed in further studies for better understanding of the gene regulatory network, molecular mechanisms of AD, developing new therapeutic approaches, future studying of miRNA function and regulation and their potential as diagnostic biomarkers for AD. However, this project is a bioinformatics analysis study based on the high throughput data and is not derived in our laboratory, but a large number of experimental studies confirm that the pathways and genes which were involved in AD are supported.

## Methods

We have detected miRNA and their target genes in AD as well as the Gene Ontology and their signaling pathways. First, we detected differentially expressed genes (DEGs) by meta-analyzing six gene microarray data sets, for the differentially expressed miRNAs (DEmiRs), a miRNA expression profile could be detected. Then using the Cytoscape 3.2.1^57^ software, DEGs and DEmiRs in order to visualize and draw the miRNA-gene interaction network. Furthermore, we calculated the active hubs and their immediate neighbors in our miRNA-gene network; we obtained the potential active miRNAs and target genes in AD. Last but not least, using the ClueGO v2.2.5 plugin^58^ in Cytoscape 3.2.1, we detected the pathways of our top miRNA-target genes and therefore, the significant pathways involved in AD (Fig. 2) were revealed. The summary of the overall study process has been shown in the diagram supplementary Figure S1.

### Microarray analysis and availability

Seven microarray data sets of human AD up to 31 December 2016 according to our criteria (Fig. 1) have been selected that are available in the public repository: NCBI Gene Expression Omnibus (GEO): GSE1297, GSE4757, GSE5281, GSE12685, GSE28146, GSE16759, in which GSE16759 was used for its miRNA expression profile^47,59–64^ and totally 264 samples (113 control and 151 cases) were analyzed in this study which their details have been mentioned in Table 1. Brain samples of each data sets were collected from Research Institute of Alzheimer’s disease in the USA; so, descriptions and categorization of the donor samples details, including the mean age, gender, and brain regions were listed in supplementary Table S9. These seven data sets were independently generated using different protocols. The quality control and normalization of our array data have been done with affy, plier and piano packages in R^65–67^. Their output has been reported in supplementary Figure S2. Analyzing AD has been done in two groups control and AD patients using the GEO2R tool, to detect differentially expressed genes (DEGs) and differentially expressed miRNAs (DEmiRs)^68^.

### Differentially expressed miRNAs in AD

High-throughput techniques to investigate miRNA expression in AD have thus far rarely been used; we found only one miRNA microarray data set in GEO (GSE16759), that studied both miRNA and mRNA expression in AD patients and controls^47^.

### Meta-analysis

Different gene expression profiles may demonstrate the varied differentially expressed (DE) genes to obtain accurate gene expressions^69^; hence, we used a meta-analysis in our study. In this study, we implemented the meta-analysis by using the R package RobustRankAggreg^70^. The scores based on the P value were calculated for all seven data sets, genes and miRNA with P-values less than 0.05 and fold change ≥  1.23 were considered as DEGs. In our study, we performed a sub meta-analysis on Male and Female with four GSEs (GSE16759 because of the low sample size and GSE4757 because the unknown gender have been excluded). On the other hand, the expression profile of six brain regions and sub meta-analysis on the hippocampus and entorhinal cortex have been done to comprise the effect of brain regions on DEGs. Then region specific genes for each brain region in AD have analyzed.

### Studying of RNA-seq data

According to our inclusion and exclusion criteria, by searching the GEO and SRA (https://www.ncbi.nlm.nih.gov/sra), all available gene expression data in the context of AD have been collected (supplementary Figure S3). The GSE53697 by the Illumina HiSeq. 2500, GSE67333 by the Illumina HiSeq. 2000 platforms and GSE57152 by AB 5500xl Genetic Analyzer platforms respectively have been selected for further analysis. The quality control (FastQC) on the RNA-seq (GSE53697, GSE67333, and GSE57152) short reads have been done (supplementary Figure S4)^71,72^; hence, samples with low quality, even after trimming were omitted. Totally 20 control samples and 21 patient samples have been selected for RNA-seq data analysis. The whole descriptions of each RNA-seq donor sample detail were categorized in supplementary Table S10. Therefore, for comparing the expression profile of RNA-seq (GSE67333 and GSE57152), TopHat2 (version 2.0.8)^73^ was utilized to align the RNA short reads to the reference human genome (hg38). Then individual transcripts were assembled with cufflinks and for generating the RNA-seq DEGs, Cuffdiff, which calculates the expression and finds significant changes in samples have been done^74,75^. Also, the available analyzed expression profile of GSE53697 was used which is in the supplementary files^12^.

### Identification of miRNA-target genes

To construct the Gene Regulatory network (GRN) for miRNA-target genes based on gene expression and DEmiRs, all of them were integrated and visualized in Cytoscape 3.2.1. In this regard, we used cyTargetLinker plugging in Cytoscape 3.2.1 to draw the miRNA-target genes network^13^. After that, an organic layout was applied on the network, all of them except Homo sapiens filtered. Furthermore, node size and color have been done on the miRNA-target genes network based on logFC for all of them.

### Identification of potential active miRNA-target genes in AD

Traced networks often have a complex structure, even if it is distilled into the smaller network. For solving these problems and analyzing the network easily, Cytoscape 3.2.1 conspicuously helps us. By centiscape plugging in that we could find the hub nodes in miRNA- target genes network. In this analysis, we calculate degree-closeness and degree-betwenness for all nodes, edges and detected the potential active miRNA node. Then we continued the study^14^. In order to determine the validated miRNA target genes as compared to predicted ones that were studied in this analysis, miRTarBase^76^, TarBase v.8 ^77^and miRWalk 2.0^78,79^ databases have been utilized.

### Enriched gene ontology and pathways analysis

To identify the biological processes and their pathways in the miRNA-target genes and gender specific genes; also, molecular function of each region specific genes, the ClueGO v2.2.5 plugin of Cytoscape 3.2.1 was used^58^. In the advanced statistical option in ClueGo v2.2.5 plugin, Two-sided hypergeometric test to calculate the importance of each term was selected and Bonferroni step-down, and Kappa score = 0.4 were used for P value correction. In this part of our study, we detect the KEGG pathways of all genes that were interacted by hub node. In our previous study in the context of network biology, we utilized ClueGO v2.2.4 plugin for visualizing the functionally related gene by network and showed the significant results based on Bonferroni step down correction and kappa score threshold^80^.

### Clustering gene expression data

Clustering of DEGs was done using the R packages gplots, RcolorBrewer which we have visualized it^81,82^. In this part of our study, we used hierarchical clustering, which is a powerful method for analyzing high throughput expression data. R calculated the similarity between genes in each data sets and showed the expression value by colors then clustering them.

## Electronic supplementary material

Supplementary Information

Supplementary Dataset 1
