# Supplementary material for: Analysis of microRNA and Gene Expression Profiles in Alzheimer’s Disease: A Meta-Analysis Approach
Source: Sci Rep. 2018 Mar 19;8:4767. doi: 10.1038/s41598-018-20959-0 (PMC5859169; doi:10.1038/s41598-018-20959-0)
Supplement: Supplementary file 1 — Supplementary Information [file 41598_2018_20959_MOESM1_ESM.pdf]

Analysis of microRNA and Gene Expression Profiles in Alzheimer's Disease: A Meta-Analysis Approach

Shirin Moradifard[1,+], Moslem Hoseinbeyki[1,+], Shahla Mohammad Ganji[1,*], Zarrin Minuchehr[1,*]

List of items:

Supplementary Table S9: Evaluation of Patients Data.

Supplementary Table S10: Patients RNA-seq Data.

Supplementary Figure S1: The summary of the overall study process.

Supplementary Figure S2: Quality control and normalization of array data.

Supplementary Figure S3: Data set selection flow chart for RNA-seq analyzing.

Supplementary Figure S4: The quality control on RNA-seq data.

Supplementary Table S9: Patients Data

GSE ID	Gender (control/case)	Brain region	Sample collection (country/city)	Mean age (control / AD)
**GSE28146**	6M,2F/ 6M, 16F*	Hippocampus (HP)	USA/ Lexington[1]	86.3 / 86.8
**GSE4757**	Unknown	Entorhinal cortex (EC)	USA/ Phoenix[2]	84.7 / 80.1
**GSE1297**	7M,2F/6M,16F	hippocampus	USA/Lexington[3]	85.3 / 85.8
**GSE12685**	4M,4F/3M,3F	Frontal cortex	USA/ Los Angeles[4]	88.4 / 91
**GSE5281**	53M,21F/50M,37F	EC, HP, medial temporal gyrus, posterior singulate, superior frontal gyrus, primary visual cortex,	USA/ Phoenix[5]	80 / 80
**GSE16759**	1M,3F/1M3F	Parietal lobe	USA/ Los Angeles[6]	85 / 91

Table S9. Evaluation of Patients Data. * F: Female, M: Male. [1] Brain Bank of the Alzheimer's Disease Research Center at the University of Kentucky. [2] The Arizona ADC, the Duke University ADC and the Washington University ADC. [3] Brain Bank of the Alzheimer's Disease Research Center at the University of Kentucky. [4] Clinical Core of the ADRC. [5] Washington University, Duke University, and Sun Health Research Institute. [6] the USC Alzheimer's Disease Research Center (ADRC).

Supplementary Table S10: Patients RNA-seq Data

GSE/SRA ID	Gender (control/case)	Brain region	Sample collection area	Mean age (control / AD)
**GSE53697 / SRP034831**	Unknown	BA9, which is part of the dorsolateral prefrontal cortex	Mount Sinai Brain Bank / USA	79 / 90.66
**GSE67333 / SRP056604**	2F, 2M / 3F, 4M	hippocampi	Branner Sun Health Research Institute / USA	83.7 / 83.5
**GSE57152 / SRP041534**	8 M / 8 M	superior temporalis gyrus	Netherland Brain Bank (NBB) / Netherland	81 / 85

Table S10. Evaluation of RNA-seq Patients Data. * F: Female, M: Male.

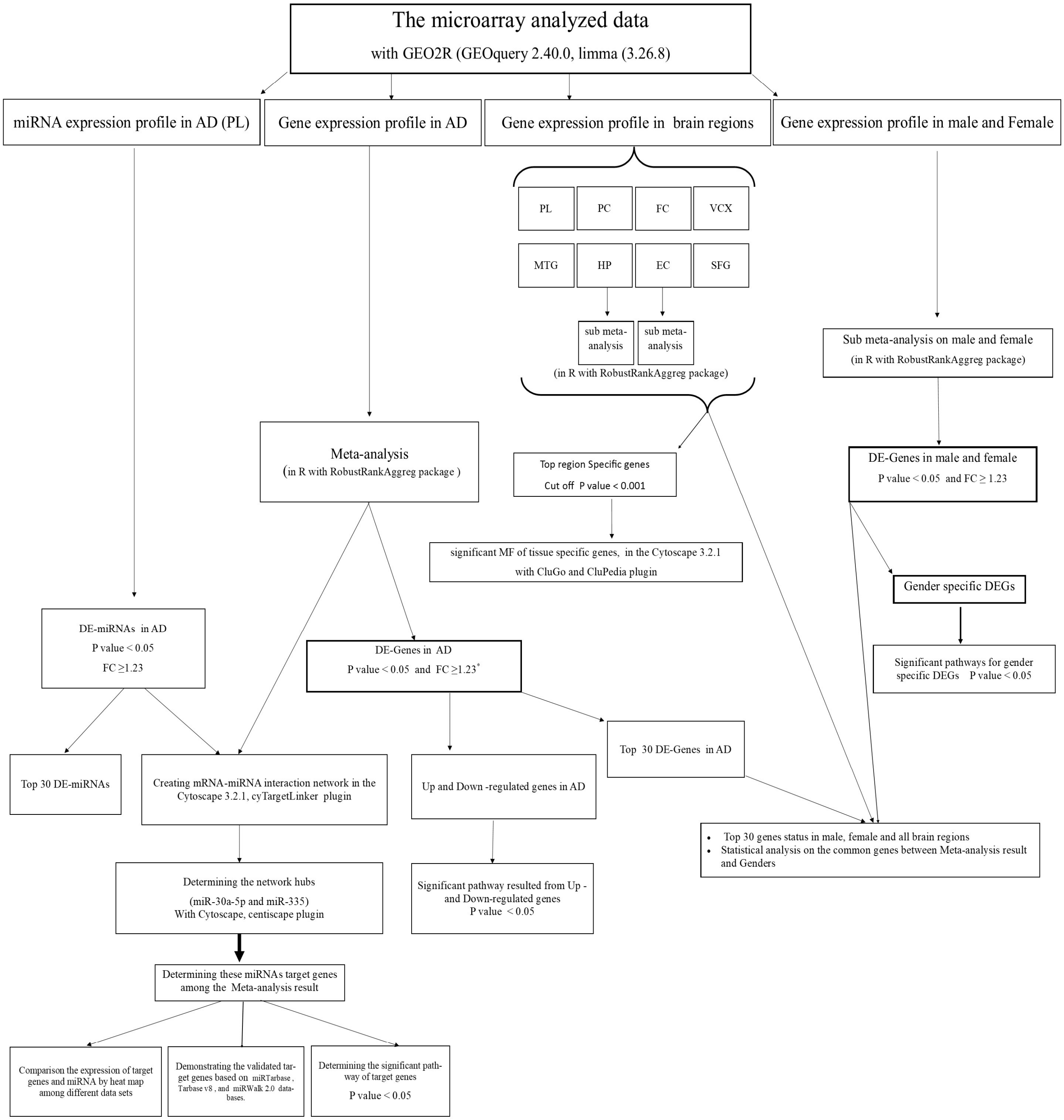

Supplementary Figure S1: The summary of the overall study process on microarray data. The summary of our study process, besides the details, tools and plugins were used, in this diagram has been shown. * demonstrated the FC > 1.23 and < 0.81.

a) Boxplots after normalization

GSE1297

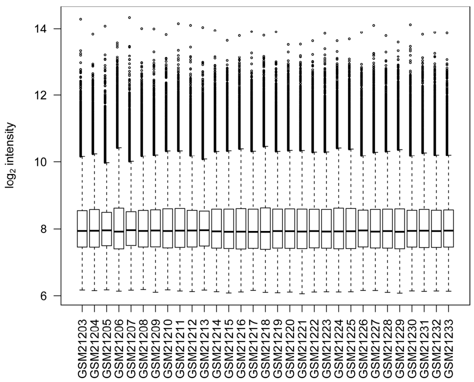

GSE4757

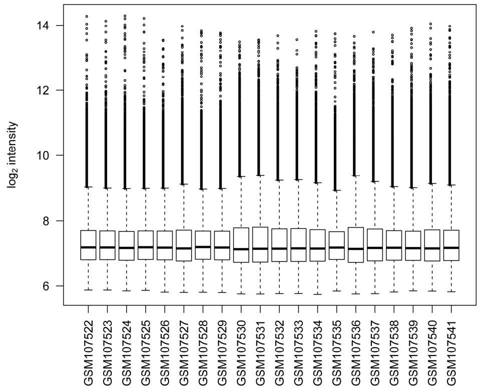

GSE5281

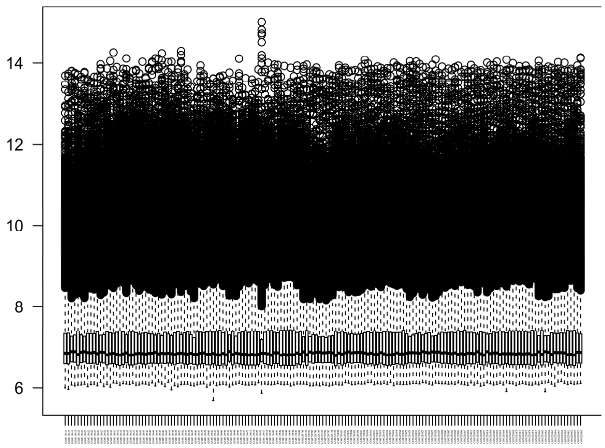

GSE12685

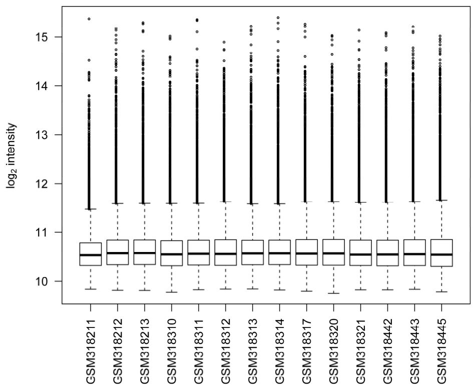

GSE16759

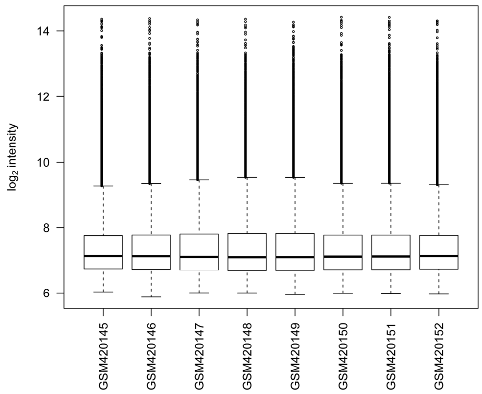

GSE28146

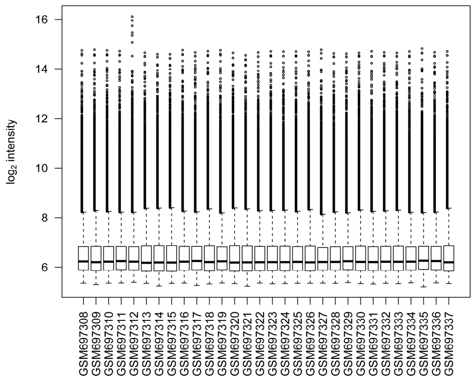

b) Density histogram after normalization

GSE1297

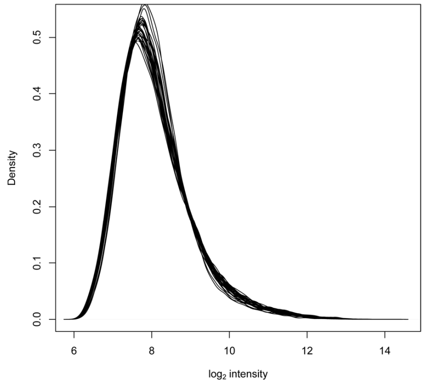

GSE4757

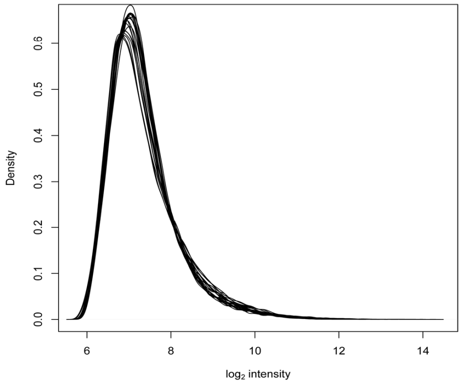

GSE5281

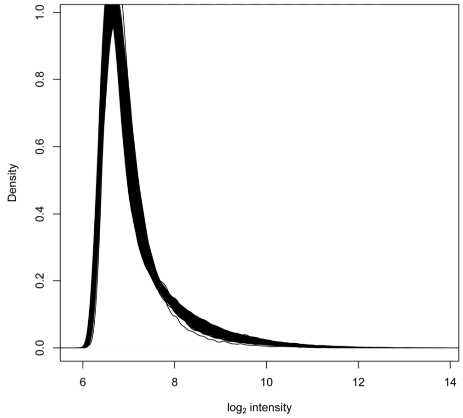

GSE12685

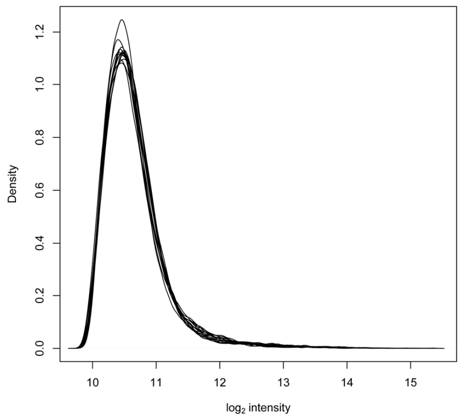

GSE16759

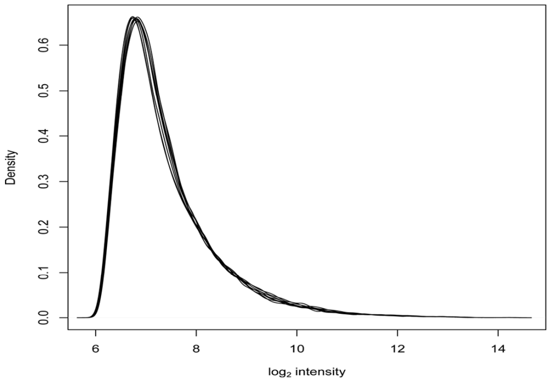

GSE28146

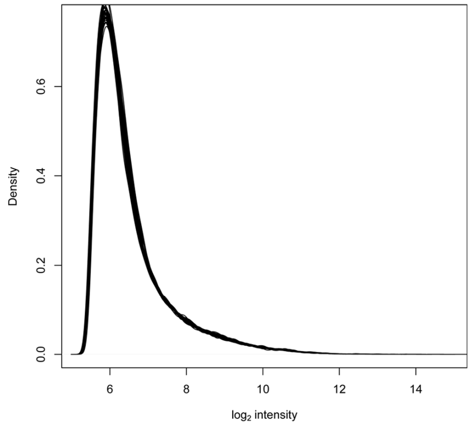

C) Relative Log Expression (RLE) Plot

GSE1297

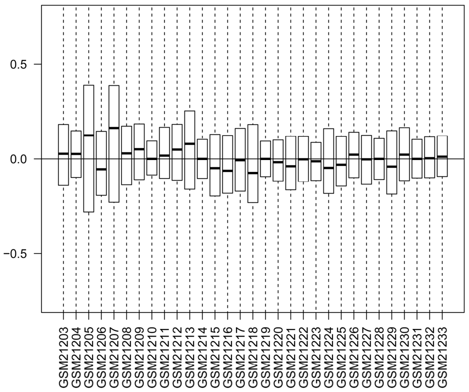

GSE4757

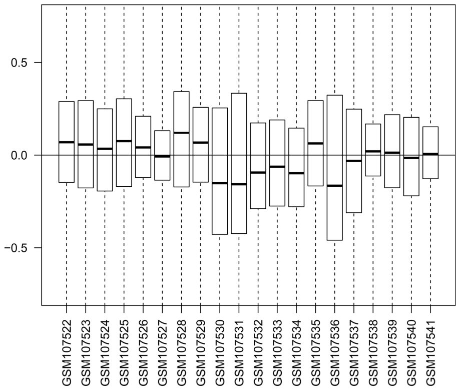

GSE5281

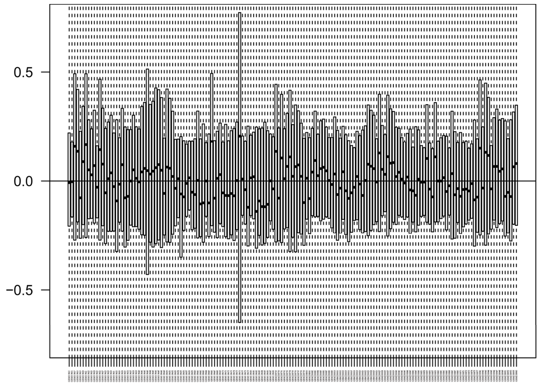

GSE12685

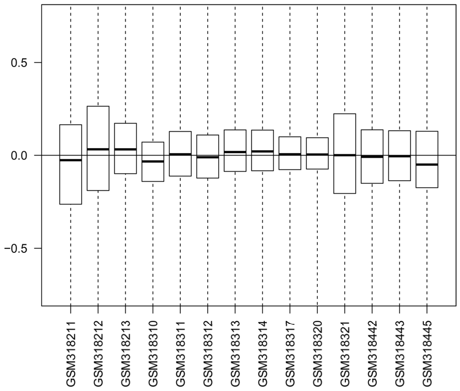

GSE16759

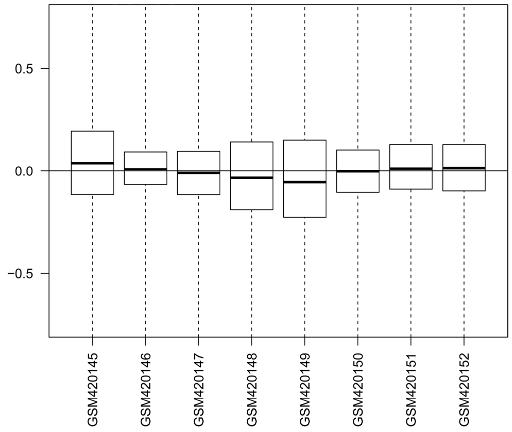

GSE28146

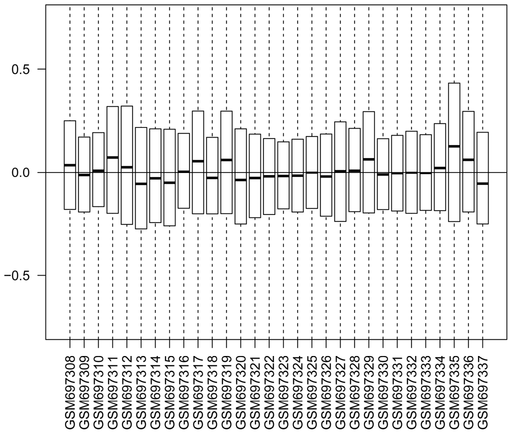

d) RNA degradation plot

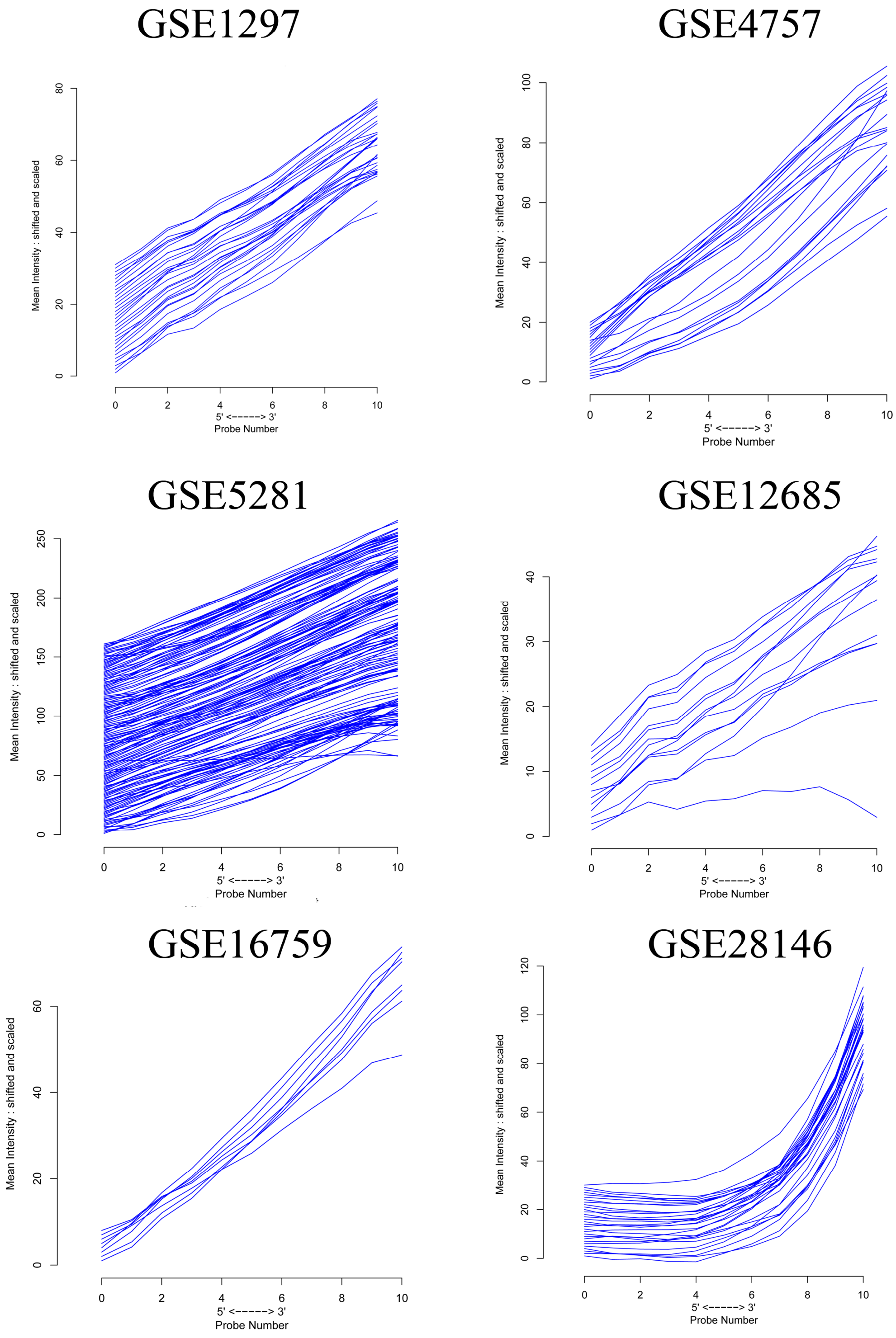

Supplementary Figure S2: Quality control and normalization for affymetrix GSE data. a) Box plots showed the normalized data in all GSEs. b) Density histogram plots, displays the frequency distribution of values in data sets. c) Relative Log Expression (RLE) plots, demonstrated the expression value of each array. d) RNA degradation plots showed the RNA quality of all GSEs.

Data set selection flowchart in RNA-seq

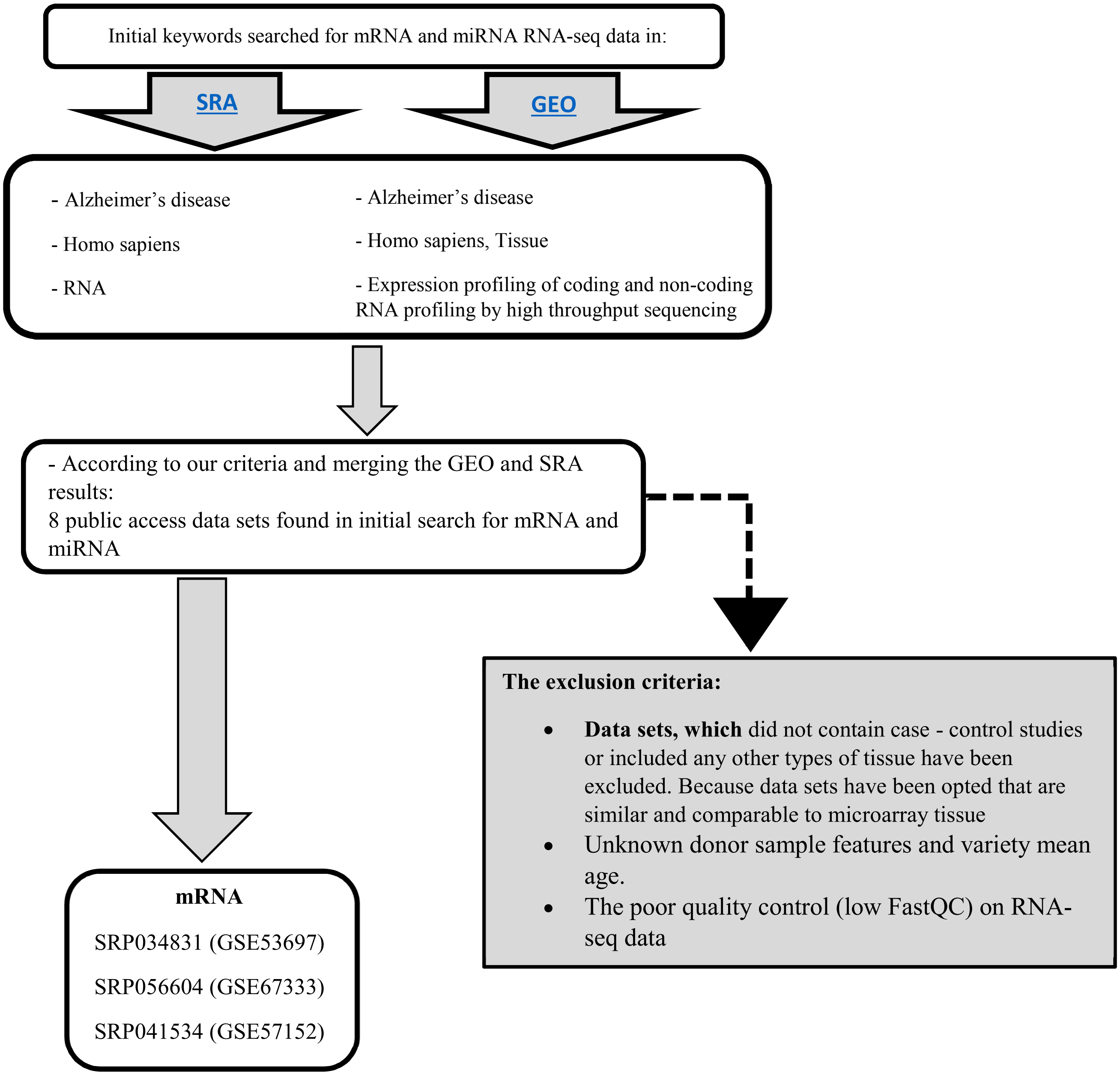

Supplementary Figure S3: Data set selection flow chart for RNA-seq analyzing. According to our criteria three RNA-seq data sets (GSE53697, GSE67333 and GSE57152) have been selected that were comparable with microarray data were evaluated.

a. b.

AD - GSE53697- SRR2422933

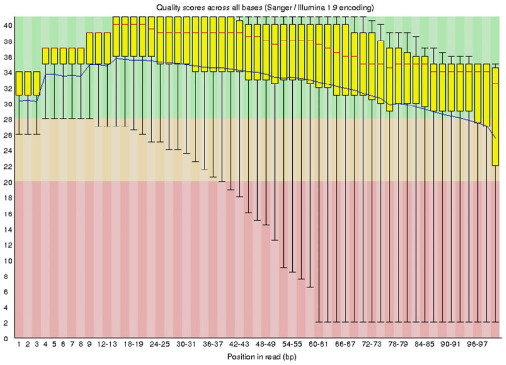

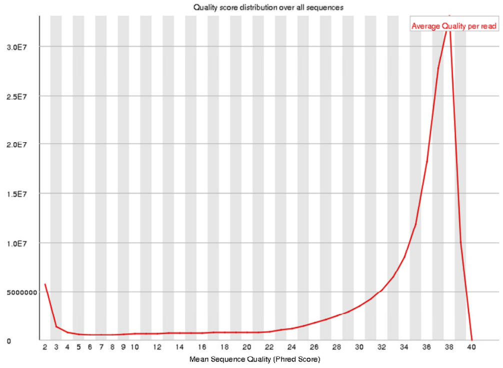

Control - GSE53697- SRR2422925

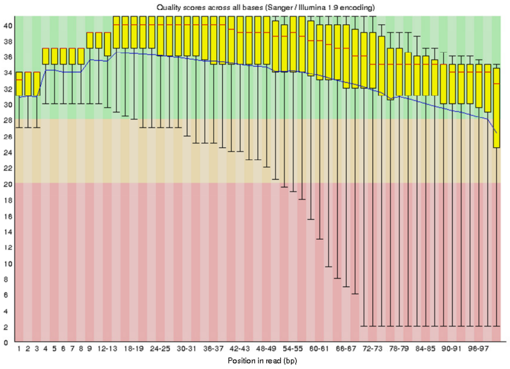

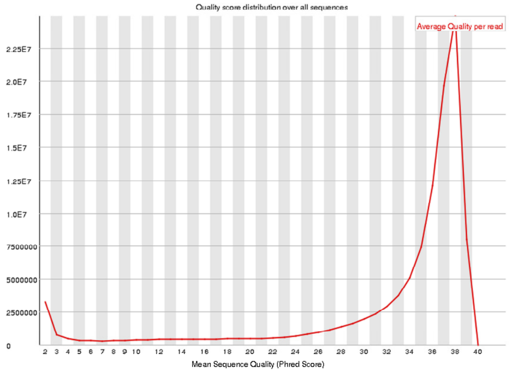

AD - GSE67333 - RR1931812

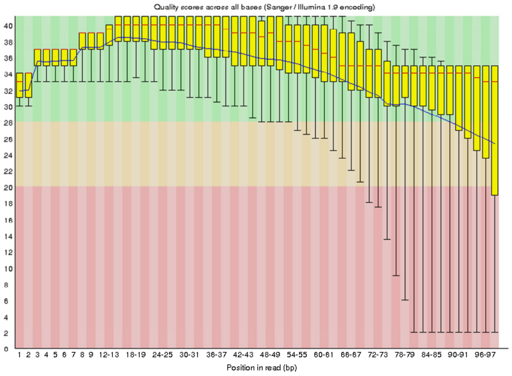

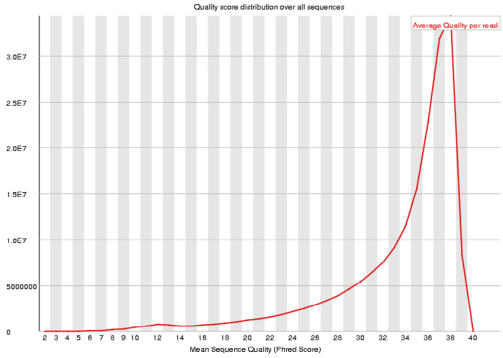

Control - GSE67333 - SRR1931818

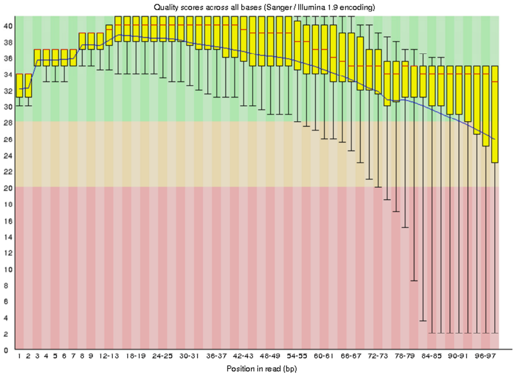

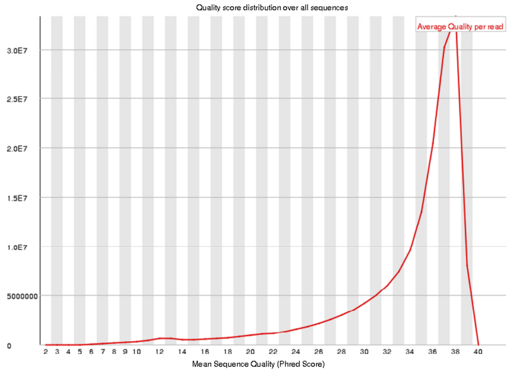

c.

AD - GSE57152- SRR1265147

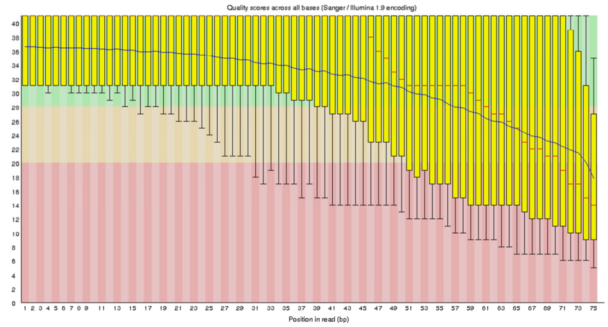

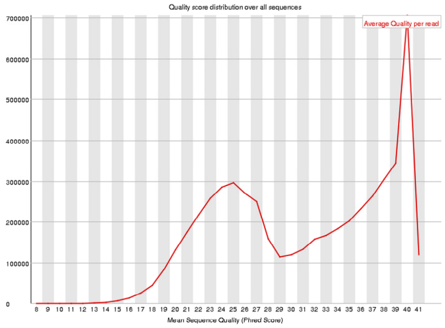

AD - GSE57152- SRR1265147 -Trimmomatic

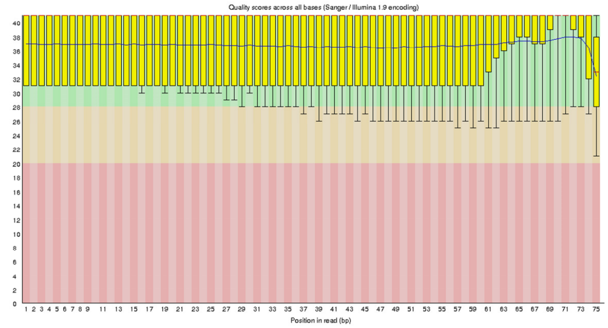

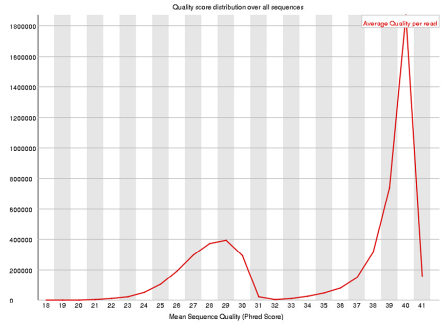

Control - GSE57152- SRR1265244

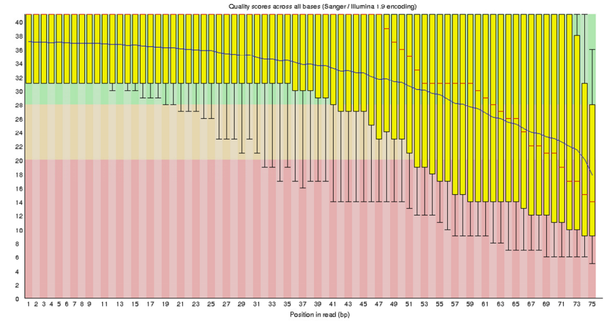

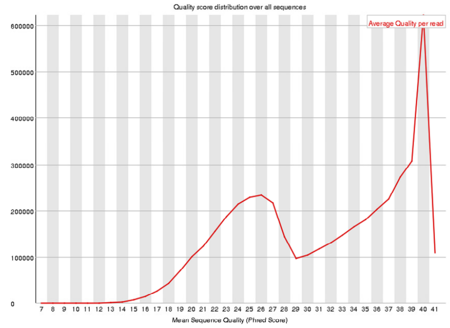

Control - GSE57152- SRR1265244 - Trimmomatic

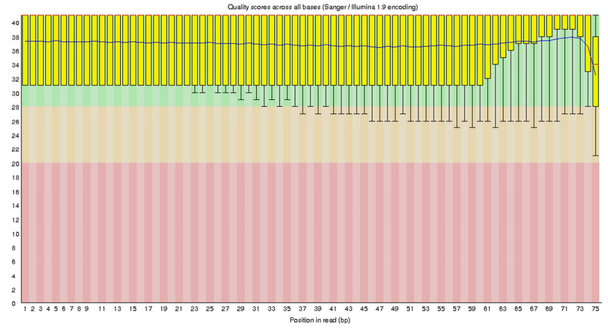

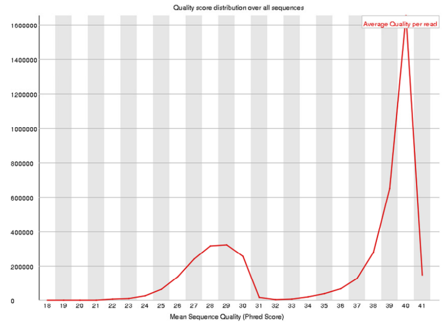

Supplementary Figure S4: The quality control on RNA-seq data. This is demonstrated the FastQC results on the GSE53697, GSE67333 and GSE57152 data sets. The a) per_base_quality and b) per_sequence_quality graphs showed the sequence quality in the AD samples (case) and healthy samples (control) which randomly selected. c) The quality control result for two selected samples of GSE57152 before and after trimmomatic statue.
